# Why They Speak Up (or Don’t): Reasons For and Against Cybergrooming Disclosure Among Adolescent Victims

**DOI:** 10.1007/s10964-025-02192-x

**Published:** 2025-05-12

**Authors:** Catherine Schittenhelm, Christine Weber, Maxime Kops, Sebastian Wachs

**Affiliations:** 1 Institute of Education, University of Münster, Münster, Germany; 2 Bundeskriminalamt, Wiesbaden, Germany

**Keywords:** Cybergrooming, Online grooming, Sexual victimization, Adolescence, Disclosure

## Abstract

The ubiquitous use of information and communication technologies among adolescents carries the risk of exposure to online victimization during this vulnerable stage of development, including cybergrooming as a form of sexual victimization. Although established in traditional abuse research, studies on disclosure processes in the specific context of cybergrooming victimization are still pending. The present study exploratively investigated reasons for and against disclosure following cybergrooming victimization in the subsample of *n* = 400 victims (44.1%; *M*_age_ = 15.48 years, girls: 57.5%) from *N* = 908 adolescent participants. Most victims disclosed to someone (86%), with peer disclosure being more frequent (73%) than disclosure to adults (55%). Findings indicated differences of small effect sizes in reasons for and against disclosure depending on the confidant (peers vs. adults; for example, the reporting of similar experiences by others was more relevant in peer disclosure). However, gender had almost no influence on the assessed reasons. In structural equation models, latent factors of reasons against, and intra- and interindividual reasons for disclosure predicted peer and adult disclosure to varying degrees, with reasons against disclosure being the most predictive in both cases. Disclosure to adults could be better explained than disclosure to peers (*R*^2^_peers_ = 28.6%, *R*^2^_adults_ = 46.9%). In open-ended items, participants provided further reasons, which were grouped into categories (e.g., help-seeking, warning/prevention, fear of bullying/social exclusion). Practical implications like the aspired congruence between reasons for disclosure and confidants’ reactions, and limitations such as the neglect of the processual character of disclosure are outlined.

## Introduction

In the digital age, various aspects of adolescent life have shifted from the analog space to the digital world. The abundance of diverse online platforms – ranging from social media and online gaming to e-learning – enables adolescents to engage in information seeking, entertainment, education, and social interaction online. In short, their psychosocial development unfolds substantially in a digital environment (Senekal et al., 2023). Despite evident benefits of digital technology use in multiple areas of development (Haddock et al., 2022), it also harbors developmental risks for young people as various forms of victimization have likewise migrated to the digital world – for example, cyberbullying (Li et al., 2024), online hate speech (Castellanos et al., 2023), and cybergrooming (Ringenberg et al., 2022). While all forms of online victimization warrant attention, cybergrooming serves as a compelling example for examining young people’s victimization experiences. This includes disclosure processes, referring to victims confiding in third parties about their victimization. Disclosure represents a critical factor influencing the course of victimization and the initiation of therapeutic and legal steps (e.g., Rau et al., 2016). Key questions about cybergrooming victimization disclosure processes are whether, why, and to whom victims disclose. However, these questions have hardly been addressed in the specific context of cybergrooming victimization. Yet, insight into factors that shape the victims’ decision for (non-)disclosure considering different confidants is crucial for developing support systems. Hence, this study aims to contribute to this endeavor by shedding light on adolescents’ reasons for and against cybergrooming disclosure to peers and adults.

### Cybergrooming Victimization in Adolescence

Cybergrooming, also known as online grooming, refers to the process of adults using information and communication technologies to influence children and adolescents to establish sexual contact (Wachs et al., 2012), e.g., cybersex, obtaining child sexual exploitation material, or offline sexual contact. In comparison to other types of sexual victimization (e.g., sexual solicitation, sexting coercion; Ray & Henry, 2025), perpetrators of cybergrooming systematically establish relationships with the minor victims, whereby the duration and depth of the contact can vary (Van Gijn-Grosvenor & Lamb, 2021). This process involves perpetrators using various manipulative grooming strategies, including deception (such as lying about age or shared interests), gift giving (like offering money or material goods in exchange for pornographic material), and sexualization (such as complimenting to introduce sexual topics) (Gámez-Guadix, De Santisteban, et al., 2021). These strategies also commonly aim to maintain the victim’s secrecy during the grooming process, for instance, by manipulating the victim into perceiving the situation as an exclusive and unique relationship that requires protection from others, or by threatening to release sexually explicit material sent by the victim if they disclose the situation (Moosburner et al., 2025; Ringenberg et al., 2022).

In summary, cybergrooming refers to a psychologically manipulative process that may lead to online and/or offline sexual abuse (e.g., Webster et al., 2012); however, it should not be equated with the latter. Instead, sexual abuse can be considered a proximate outcome of the cybergrooming process. However, both cannot be distinguished as separate successive stages; for example, sexually abusive acts, such as the exchange of pornographic material, can be utilized by the perpetrator to consolidate the sexually exploitative relationship through further grooming efforts. This lack of clear separability between the grooming process and sexual abuse is also reflected in empirical research, as measuring cybergrooming victimization often includes items related to the exchange of sexually explicit material, offline abuse, and sextortion (e.g., Bergmann & Baier, 2016; Gámez-Guadix et al., 2018). Therefore, research on related phenomena such as online sexual solicitation (Gemara et al., 2025), Internet child sexual abuse (Katz et al., 2021), and sextortion (Wolak et al., 2018) is highly informative in the context of cybergrooming.

Adolescents may be particularly at risk for experiencing cybergrooming victimization, as adolescence represents a developmental phase in which young people increasingly use the Internet for social interaction (Feierabend et al., 2022), explore their sexuality on- and offline (e.g., Eleuteri et al., 2017), and seek extrafamilial social relationships (Giordano, 2003). Reported victimization prevalence rates among adolescents vary across studies because of, for example, different reference periods. A current systematic review indicated that most reported prevalence rates range between 10 and 20%, with most relating to a reference period of up to one year (Schittenhelm et al., 2024). However, in longitudinal studies examining longer periods, overall prevalence rates increased to 25–35% (e.g., Gámez-Guadix et al., 2023). Thus, a substantial number of young people fall victim to cybergrooming, which can adversely impact their mental well-being and social development. Specifically, cybergrooming victims reported higher depressive symptoms and anxiety (Gámez-Guadix, De Santisteban, et al., 2021), reduced health-related quality of life (Calvete et al., 2020), and adverse social consequences (e.g., peer and intrafamilial problems; Whittle et al., 2013). However, research explicitly focusing on cybergrooming victimization outcomes is limited so far. Research on related phenomena, such as pressured sexting and threats to post sexually explicit material, has shown a range of severe potential outcomes for victims. These include post-traumatic stress (Patel & Roesch, 2022), non-suicidal self-harm (Wright & Wachs, 2024), and suicidal ideation, suicide plan, and attempt (Srivastava et al., 2022).

### Disclosure Processes Following Sexual Victimization

In the context of victimization, disclosure refers to victims confiding in someone about their victimization experiences. Importantly, it does not represent a single event but rather a complex process that involves the victims evaluating whether to tell, withhold, or even recant the victimization experience, which confidants to disclose to, and how confidants react to the disclosure (e.g., Brattfjell & Flåm, 2019). Disclosure is crucial from two perspectives. Focusing on the victims, disclosure can impact the course of the victimization by ending it and preventing revictimization (e.g., Rau et al., 2016). For instance, support or advice from trusted confidants can be crucial for victims to escape an abusive situation, especially for those who were not able to do so on their own. Further, disclosure can initiate therapeutic intervention if indicated and has the potential to mitigate adverse mental health outcomes of sexual victimization (e.g., McTavish et al., 2019). For the beneficial effects of disclosure, the confidants’ reactions – e.g., helpful versus non-helpful (Easton, 2019) – are relevant; however, this falls beyond the scope of the present study. Focusing on perpetrators, disclosure is critical to initiate legal steps (Rau et al., 2016). By reporting to law enforcement authorities, perpetrators can be identified and prosecuted. As a result, further victims may be identified, and other young people may be protected from victimization.

Despite the potential benefits of disclosure, a substantial proportion of young people who become victims of sexual violence do not disclose their experiences to anyone (e.g., Maschke & Stecher, 2021; Mennicke et al., 2022). Furthermore, in the event of disclosure, peers are preferred over adults as confidants (e.g., Priebe et al., 2013). Consistent with this, cybergrooming research revealed that approximately two-thirds of victims disclose to someone, with peers being the main confidants (Juusola et al., 2021; Villacampa & Gómez, 2017). This preference for peer disclosure among young people is evident across different types of sexual victimization – online and offline – and different countries (e.g., Gemara et al., 2025; Mapes & Cavell, 2021). However, research suggests that victims often confide in more than one person in a gradual process in which peer disclosure precedes and, for example, encourages victims to confide in adults. This, in turn, is likely to initiate contact with formal support and authorities (Manay & Collin-Vézina, 2021).

### Reasons For and Against Disclosure

To understand why some victims choose to confide in someone while others do not, knowing their reasons for and against disclosure is essential. However, research on this issue in the context of cybergrooming victimization is scarce. Initial studies revealed that many victims did not perceive the situation as serious, other reasons against disclosure were insufficient courage to tell, a lack of anticipated interest of others or help through disclosure, and feelings of shame and guilt (Juusola et al., 2021; Villacampa & Gómez, 2017). Research on related phenomena further discussed self-blame and embarrassment (Katz, 2013) as well as fear of dissemination of intimate material by the perpetrator (Katz et al., 2021), adverse reactions such as blame or disbelief (Gemara et al., 2025), and getting in trouble or being punished (Wolak et al., 2018) as barriers to disclosure. Briefly addressed reasons for disclosure of cybergrooming experiences included others bringing the subject up or asking directly, as well as feelings of anxiety and support seeking (Juusola et al., 2021).

In traditional abuse research, the body of literature regarding reasons for and against disclosure following sexual victimization, especially child sexual abuse (CSA), is more comprehensive. Here, multiple factors that prompt or hinder victims from confiding in others have been identified, which allowed for the organization of these factors by building on a socio-ecological framework (e.g., Alaggia et al., 2019; Zinzow et al., 2022). The socio-ecological model was introduced to describe how individuals develop within interconnected layers of their environment – from relationships with their direct surroundings to the cultural context – and illustrates the interplay of individual and multi-layered environmental factors (Bronfenbrenner, 1979). According to this person-in-environment approach, disclosure processes are shaped by the interaction of intrapersonal, interpersonal, and contextual factors. While intrapersonal factors arise from within the victim (e.g., emotional responses) and interpersonal factors result from the victim’s relationships and interactions with actors in their direct environment (e.g., anticipated reactions to disclosure), contextual factors refer to the cultural context determined by community and societal norms and attitudes (e.g., laws, gender roles) (e.g., Collin-Vézina et al., 2015). Based on this categorization, Table 1 depicts examples of intrapersonal (e.g., Jacques-Tiura et al., 2010; Schaeffer et al., 2011), interpersonal (e.g., Lemaigre et al., 2017; McElvaney et al., 2014), and contextual reasons (e.g., Alaggia et al., 2019; Collin-Vézina et al., 2015) for and against disclosure.

**Table 1:** Reasons for and against disclosure from a socio-ecological perspective

	Intrapersonal	Interpersonal	Contextual
Reasons for disclosure	Emotional support-seeking	Reporting of similar experiences by others	Environment generally addressing the topic
	Confusion about own feelings	Being directly approached or asked about it	Solid infrastructure of available services
	Wish for the victimization to end	Supportive parent-child relationship	
Reasons against disclosure	Feelings of shame, self-blame, guilt, and embarrassment	Threats, blackmail, or interdictions by the perpetrator	Stigmas associated with victimization
	Affection for the perpetrator	Fear of disbelief or punishment	Taboo of sexuality

Please note that this does not represent an exhaustive overview but is only intended to illustrate the categorization into intrapersonal, interpersonal, and contextual factors. The term ‘interdiction’ refers to the perpetrator forbidding the victim to tell anyone about the victimization experience, or more specifically, in the context of cybergrooming, about the online contact

### Differences in Reasons For and Against Disclosure

Previous research indicates that disclosure decisions involve diverse reasons for and against it. However, it is conceivable that several factors influence how pronounced these reasons are in individual disclosure processes. For instance, gender might influence the strength of different reasons. Traditional abuse research suggests that fears associated with gender roles and stigmatization act as barriers to disclosing sexual victimization for men in particular (e.g., Easton et al., 2014). While quantitative studies on gender differences are still rare, one study on disclosure following CSA found no gender differences in young adults in reasons against disclosing to formal recipients (e.g., police, health care providers) and only very few gender differences in reasons against disclosing to informal confidants (e.g., peers, family members; Okur et al., 2020). Further, differences in the strength of reasons might occur based on the specific confidant to whom a victim considers disclosing. Building on traditional abuse research, victims disclose to different confidants for different reasons after sexual abuse (Manay & Collin-Vézina, 2021); when confiding to peers, adolescents may primarily aim for emotional support and help to tell an adult, while in adult disclosure, the wish for the abuse to stop and for assistance in seeking formal support might be particularly relevant. Further, victims of online sexual solicitation explained their preference for peer disclosure by perceiving their peers to be more closely related to experiences of sexuality and sexual abuse, to be more proficient with technology, and fearing social media restrictions as well as more negative reactions from their parents, such as exaggerations and blame (Gemara et al., 2025).

To summarize, research on disclosure following cybergrooming victimization or related phenomena (e.g., Gemara et al., 2025; Juusola et al., 2021) provided valuable initial insights into reasons for and against disclosure. Nevertheless, the reasons in favor of disclosure have been mostly overlooked by focusing on reasons against it. Considering findings from traditional abuse research, the reasons for disclosure could be just as manifold as the reasons against it. Furthermore, previous analyses of reasons were qualitative or rudimentary quantitative. Methodologically, this research did not go beyond frequency analyses of dichotomously conceptualized reasons, precluding statements about how pronounced different reasons are. Lastly, factors influencing reasons for and against disclosure, such as gender or the intended confidant, have hardly been addressed. Therefore, the present study served a broader and more in-depth analysis of whether further reasons can be identified and whether these reasons differ between and within persons. Addressing these research gaps regarding disclosure of cybergrooming victimization is crucial: First, knowledge about reasons for and against disclosure can be used to create an environment facilitating the initiation of disclosure processes for victims. Second, that knowledge is needed to raise general awareness of how to react appropriately to disclosure as a confidant (Edwards et al., 2022). Confidants’ reactions should not reflect the victim’s contemplated reasons against disclosure, for instance, invalidation or social media restrictions and confiscation of technological devices. Negative disclosure experiences may reduce the likelihood of the process progressing – e.g., reporting to authorities – and future disclosure in the event of re-victimization (Easton et al., 2014; Jackson et al., 2017). Further, they may also harm victims’ well-being (McTavish et al., 2019).

## The Present Study

Adolescence marks a key developmental stage for digital socialization, including the exploration of sexuality and the development and maintenance of relationships online. During this vulnerable phase, young people are facing the risk of cybergrooming victimization, posing a potential threat to their psychosocial development and mental well-being. Disclosure has the potential to mitigate negative consequences of victimization and initiate prosecution of the perpetrators. Yet, research on disclosure processes following cybergrooming victimization among adolescents remains severely limited. To this end, the first objective of the present study was to examine the frequency rate of disclosure and chosen confidants among cybergrooming victims. As knowledge about reasons for and against disclosure is crucial to facilitate the disclosure process and promote positive disclosure experiences for victims, the second objective was to gain deeper insights into victims’ reasons for and against disclosure. Building on abuse research, it was expected that in the specific context of cybergrooming victimization, the disclosure process is likewise shaped by various intrapersonal, interpersonal, and contextual reasons for and against disclosure. The third objective was to investigate whether these reasons vary in strength based on the victims’ gender and the potential confidant (i.e., peers, adults). Finally, the fourth objective was to examine the association between reasons for and against disclosure and actual disclosure behavior.

## Methods

The present study was preregistered on October 24th, 2024, with OSF Registries (10.17605/OSF.IO/U8YBR).

### Sample and Data Cleaning

Data was collected by a market research company specializing in families and children (KB&B), whose underage panelists can only participate in studies with their parents’ consent. KB&B conducted an initial quality check based on participants’ response behavior and survey completion time. Subsequently, a dataset of *N* = 1014 participants was provided. Participants who exhibited highly consistent response behavior (*n* = 16) were excluded from this dataset, resulting in *N* = 998 participants. In the next step, after examining items in more detail and excluding two items from analyses (see Analytical Approach), *n* = 84 participants were identified as multivariate outliers and removed. Since the case number of other genders was very low (*n* = 6) and gender was included as a control variable in all analyses, all participants with a gender other than female or male were excluded. The final sample size after stepwise data cleaning was *N* = 908 (see Supplementary Material Fig. 1 for a more detailed representation of the data cleaning process). All participants who completed the survey were awarded bonus points by KB&B. Table 2 presents sample characteristics for the final total sample and the subsample of victims.

**Table 2:** (Sub)Sample characteristics

(Sub)Sample	*N* (%)	*M*_Age_ (*SD*)	Range_Age_	Gender (%)	School (%)
Total sample	908 (100)	15.43 (1.11)	14–17	female: 52.4 male: 47.6	*Gymnasium*: 47 *Realschule*: 21.5 *Hauptschule*: 6.7 Other: 24.8
Victims	400 (44.1)	15.48 (1.11)	14–17	female: 57.5 male: 42.5	*Gymnasium*: 44.8 *Realschule*: 21.7 *Hauptschule*: 7.7 Other: 25.8

Age is given in years. After primary school, the German school system divides into different school forms, with the highest qualification obtained at the *Gymnasium*

### Procedure

The study received approval from the ethics board of the Institute of Education at the University of Münster, Germany. The survey was conducted online. Before consenting, participants were informed about cybergrooming as the survey’s subject, the full anonymization of their data, and the option to withdraw from the study at any time. Two questionnaire versions were developed: a victim and a non-victim version. If participants indicated that they had experienced at least one of the described situations related to cybergrooming, the victim questionnaire was administered. In addition to assessing cybergrooming victimization, reasons for and against disclosure, and disclosure behavior, further variables (e.g., evaluation of disclosure) were evaluated. However, these variables were not included in the analyses reported in this study. The victim questionnaire differed in some instructions, the specific wording of items (e.g., “I was worried that I wouldn’t be believed” vs. “I would be worried that I wouldn’t be believed”), and the assessment of actual disclosure and its evaluation compared to the non-victim questionnaire. Finally, participants received information about support services.

### Measures

The Supplementary Material (Table 1) provides an English translation of all administered items for all constructs described below.

#### Cybergrooming Victimization

Cybergrooming victimization was assessed using a short version of the Multidimensional Online Grooming Questionnaire (MOGQ; Gámez-Guadix, De Santisteban, et al., 2021) and one adapted global item (Wachs et al., 2012). The MOGQ comprises five subscales, namely deception, gift giving, interest in the victim environment, sexualization, and aggression, reflecting common strategies of cybergrooming. Items were first translated into German, and subsequently, one item per subscale was selected based on six expert ratings. To further substantiate the item selection, the five chosen items were psychometrically examined in the Spanish original data (Gámez-Guadix, De Santisteban, et al., 2021), demonstrating good model fit in a unidimensional confirmatory factor analysis (CFA) model (χ²(10) = 298.88, RMSEA = 0.037, SRMR = 0.024, CFI = 0.994, ω = 0.76; see Analytical Approach for information on CFA). Further, selected items correlated substantially with the mean value of the respective total subscale, ranging from *r* = 0.73–0.92. For each situation described by the MOGQ items (e.g., “Such an adult person offered me money or other things in exchange for photos or videos of myself”), participants were asked to indicate how often they had experienced the respective situation. To clarify the situations described for participants, exemplary short chat sequences were designed for each item (see Supplementary Material Fig. 2). Corresponding items and chat sequences were presented together, and participants were informed that this is an example of how a chat in the described situation could look. The global item asked how often a participant had any contact in total with an adult who wanted to have romantic or sexual contact with them online. It was administered to identify those participants as victims who had not experienced any of the specific MOGQ situations. All cybergrooming items were answered on a four-point Likert Scale from “Never” (0) to “5 times or more” (3). A specific reference period was deliberately not specified.

#### Disclosure Behavior

Disclosure was assessed separately for adults and peers with dichotomous items (Yes/No). In victims disclosing to adults, an open-ended question was asked about which adults were confided in.

#### Reasons For and Against Disclosure

Regardless of disclosure behavior, all participants were asked about reasons for and against disclosure. They were assessed separately for disclosure to adults and peers with six items each, guided by prior research on disclosure processes after sexual victimization (Kellogg & Huston, 1995; Lahtinen et al., 2018; Von Moy, 2012). For both reasons for disclosure (e.g., “Because someone else has reported similar experiences”) and against disclosure (e.g., “I was embarrassed”), five items could be answered on a five-point Likert scale from “Does not apply at all” (1) to “Fully applies” (5). One open-ended item each asked for further reasons for and against disclosure.

### Analytical Approach

All analyses were conducted with R (R Core Team, 2024). To make all analyses reproducible, all materials necessary are provided in an online repository: https://osf.io/aqd5k/files/osfstorage. Data analysis was conducted in two steps. First, items were examined based on *N* = 998 participants. Since one reason against disclosure exhibited comparatively low item-total correlations (*r*_peers_ = 0.37, *r*_adults_ = 0.36) and low loadings in a unidimensional CFA model of reasons against disclosure (λ_peers_ = 0.39, λ_adults_ = 0.39), it was excluded from further analyses. It is conceivable that, due to its wording (“I didn’t/wouldn’t want the contact to end.”), the item was not understood by all participants – as intended – to refer to perpetrators, but instead to the potential confidant, and therefore did not work well. Subsequently, multivariate outliers were identified based on the Mahalanobis distance. All analyses and findings from the second data analysis stage, as described and reported below, were based on data from *n* = 400 victims in the remaining total sample.

#### Categorization of Open-ended Item Responses

Responses to the open-ended question about which adults participants had disclosed to were evaluated for the first objective of the study. They were only coded by the first author, as they left no room for interpretation. For the second objective, responses to open-ended items asking for further reasons were grouped into categories. First, categories were derived based on all responses per variable (reasons for disclosure peers/adults, reasons against disclosure peers/adults), accompanied by an assignment of responses to the categories by the first author. Then, all categories were described in a codebook, based on which a second independent rater also categorized responses. The interrater reliability for each category was quantified using Cohen’s Kappa (κ). Coding discrepancies were discussed until a consensus was reached. The examples of open responses presented in the following were translated from German into English.

#### Descriptive Analyses

Differences in victimization and disclosure rates according to gender were examined via Chi-square tests. Cohen’s *h* was calculated to determine the effect size of differences between rates. For the third objective of the study, paired *t*-tests were employed to investigate differences in reasons for and against disclosure according to confidants (peers vs. adults). Welch’s *t*-tests were computed to investigate differences in reasons between female and male victims, and disclosing and non-disclosing victims, addressing the third and fourth objective. Effect sizes of mean differences were quantified using Cohen’s *d* (for paired samples).

#### Latent Variable Analyses

Latent variable analyses were performed to address the fourth objective more in-depth, using the R package *lavaan* (Rosseel, 2012). There were no missing values on closed-ended items; therefore, missing data handling was not necessary. CFAs were conducted to test for the dimensionality of constructs of interest (cybergrooming victimization, reasons for disclosure peers/adults, reasons against disclosure peers/adults). In all CFA models, maximum likelihood with robust standard errors (MLR) was used for estimation. To evaluate model fit, robust CFI (Comparative Fit Index), robust RMSEA (Root Mean Square Error of Approximation), and SRMR (Standardized Root Mean Square Residual) were considered as fit indices. Good model fit was indicated by CFI ≥ 0.95, RMSEA ≤ 0.06, and SRMR ≤ 0.08 (Hu & Bentler, 1999). Acceptable model fit was indicated by CFI ≥ 0.90, RMSEA ≤ 0.08, and the SRMR ≤ 0.10 (Bentler, 1990; Browne & Cudeck, 1992). For reliability estimation of scales, McDonald’s omega (ω; McDonald, 1999) was used, with ω ≥ 0.65 indicating acceptable reliability (Kalkbrenner, 2023).

Structural equation modeling (SEM) was employed to model associations between reasons for and against disclosure and disclosure behavior. Since structural equation models incorporated disclosure behavior as a dichotomous exogenous variable, Weighted Least Squares Means and Variance adjusted (WLSMV) estimation was applied (Beauducel & Herzberg, 2006). CFI, RMSEA, and SRMR were considered fit indices, with the same thresholds for acceptable and good model fit as used for CFA models.

## Results

### Cybergrooming Victimization Rates

Overall, 44.1% of all participants (*n* = 400) indicated for at least one cybergrooming item that they had experienced the described situation at least once and were thus classified as victims. Victimization rates showed that a higher proportion of girls than boys were victims (girls: 48.3%, boys: 39.4%; χ²(1) = 7.03, *p* = 0.008, *h* = 0.18). Overall, cybergrooming situations were mostly experienced only once or twice, while victims who experienced the situations five times or more were the minority. However, most victims (*n* = 333) had experienced at least two situations at least once, and still more than half of the victims (*n* = 244) had experienced at least three. See Supplementary Material Table 1 for descriptive statistics of all single items.

### Disclosure Rates and Chosen Confidants

Regarding the first objective, most victims (86%, *n* = 344) disclosed to someone. More specifically, 42% disclosed to both peers and adults (*n* = 167), 31% disclosed only to peers (*n* = 124), and 13% disclosed only to adults (*n* = 53). Of *n* = 201 victims who responded to the voluntary open-ended question about which adult they had disclosed to, the majority named their parents (parents: *n* = 83, only mother: *n* = 73, only father: *n* = 20). Other informal confidants, such as uncles or teachers, were mentioned rarely (in total *n* = 22). Formal confidants, namely police, therapist, and school social worker, were each mentioned only once. Taking gender into account, girls disclosed more often to peers than boys (girls: 77%, boys: 67%; χ²(1) = 4.34, *p* = 0.037, *h* = 0.22). Regarding disclosure to adults, disclosure rates were similar for girls and boys (girls: 54.3%, boys: 55.9%; χ²(1) = 0.04, *p* = 0.840).

### Reasons For and Against Disclosure

#### Closed-ended Items

To address the second objective of the study, closed- and open-ended items on reasons for and against disclosure were analyzed. Table 3 depicts descriptive statistics for all closed-ended reason items. On average, agreement with reasons for disclosure was higher than with reasons against disclosure. Regardless of the confidant, the strongest reason for disclosure was the *wish for improvement of well-being*, and the weakest was *being asked*. Among reasons against disclosure, *embarrassment* was the strongest reason, while *blackmail, threat, or interdiction by the perpetrator* was the weakest.

**Table 3:** Descriptives for closed-ended reason items and mean differences (Peers vs. Adults)

Reason		Peers *M* (*SD*)	Adults *M* (*SD*)	Mean difference	|*d*|
For disclosure	Wish for victimization to end	3.59 (1.21)	3.94 (1.21)	0.35***	0.28
	Wish for punishment of the perpetrator	3.27 (1.29)	3.53 (1.31)	0.26***	0.26
	Wish for improvement of well-being	4.24 (0.90)	4.11 (1.06)	−0.13**	0.13
	Being asked	2.95 (1.16)	2.97 (1.26)	0.02	–
	Reporting of similar experiences by others	3.60 (1.14)	3.17 (1.35)	−0.43***	0.37
Against disclosure	Embarrassment	3.33 (1.29)	3.25 (1.42)	−0.08	–
	Fear of disbelief	2.83 (1.37)	2.65 (1.37)	−0.18**	0.16
	Fear of punishment	2.50 (1.35)	2.67 (1.46)	0.17***	0.18
	Blackmail/threats/interdictions by the perpetrator	2.14 (1.28)	2.14 (1.25)	0.00	–

*** *p* < 0.001, ** *p* < 0.01. |*d*| = absolute Cohen’s *d*

#### Open-ended Items

Participants were also asked to state further reasons for and against disclosure in free text fields. In some responses, participants repeated reasons from closed-ended items. In particular, for both disclosure to peers and adults, *improvement of well-being* was re-emphasized as a reason for (e.g., “To make me feel better”, “Because it distressed me”) and *embarrassment* as a reason against disclosure (e.g., “It’s just extremely embarrassing”, “One is ashamed in front of the adults”). All derived categories that reflected reasons from closed-ended items and were therefore redundant (*improvement of well-being, embarrassment, fear of punishment, blackmail/threat/interdictions by the perpetrator*) were neglected in the following to focus on those categories that provided information beyond the closed-ended items. All further derived categories are presented and illustrated with an example in Table 4. The interrater reliability was almost perfect for each category, including non-reported redundant categories, except for two *Other* categories where it was only moderate (Landis & Koch, 1977). It should be noted that the categories are not claimed to be distinct (e.g., *exchange of experiences* could be linked to *help-seeking*) or entirely unrelated to closed-ended items (e.g., *help-seeking/competence* could include the *wish for the victimization to end*).

**Table 4:** Categories of reasons for and against disclosure from open-ended items

Reason	Category	Example	∑	κ
For disclosure (peers)	Exchange of experiences	“I just wanted to talk about whether others have experienced the same”	40	0.88
	Warning	“To warn him so that something similar will not happen to him”	24	0.91
	Help-seeking	“Because I wanted help”	24	0.88
	Safety/fear	“Feel safer”	22	0.95
	Preferring peers over adults	“She understands me and the situation better than perhaps older people”	19	0.85
	Trust	“Trust in my best friend”	14	0.82
	Encouragement	“If it happens to my friend, so that she would tell me too”	8	1.0
	Other	“I found it random and entertaining, not funny but amusing”	26	0.46
Against disclosure (peers)	Bullying/social exclusion	“That he or she would laugh at me or tease me. Or that they exclude me.”	13	0.91
	Fear (unspecified)	“My own fear”	6	0.92
	Spreading	“That it will be spread at school”	6	0.91
	Labeling	“Maybe they’ll think I’m weird”	5	0.89
	Other	“There wasn’t a good occasion”	18	0.83
For disclosure (adults)	Help-seeking/competence	“To do something about it together, for example to go to the police”	51	0.85
	Safety/protection/fear	“Safety and protection”, “Because I was scared”	22	0.95
	Trust	“Because I trust these adults”	12	0.83
	Prevention	“I don’t want him to be able to do it with any other person”	8	1.0
	Other	“To clarify the situation for myself”	21	0.56
Against disclosure (adults)	Fear (unspecified)	“I just was a bit afraid”	7	1.0
	Overreaction	“That it’s being made into a huge deal”	4	1.0
	Lack of understanding	“Sometimes they don’t understand”	4	0.89
	Other	“I’m in love with the person”	14	0.84

∑ = absolute number of mentions, κ = Cohen’s Kappa. Examples were translated from German into English

Concerning disclosure to peers, *n* = 156 victims provided further reasons for disclosure, and *n* = 43 provided further reasons against disclosure. The most frequently stated reason for disclosure to peers was the wish to share and discuss (mutual) experiences (*n* = 40). At the same time, fear of bullying, ridicule, or social exclusion was the most frequently mentioned reason against disclosure (*n* = 13). Regarding disclosure to adults, *n* = 106 victims provided further reasons for disclosure; almost half of these responses expressed the wish for or anticipation of help (*n* = 51). Only 28 participants provided further reasons against disclosure to adults. In each case, occasionally mentioned reasons not fitting into any other category were grouped into an *Other* category.

### Differences in Reasons For and Against Disclosure

#### Differences Depending on Confidants

For the third objective, mean differences between corresponding reasons (peers vs. adults) were calculated, which are presented in Table 3. For most items, there were significant mean differences of small effect size between corresponding reasons. For example, *reporting of similar experiences by others* was a stronger reason for disclosure to peers than for disclosure to adults, and vice versa for the *wish for the punishment of the perpetrator*.

Regarding open responses, some categories were only mentioned concerning peer disclosure (e.g., *encouragement*, *fear of bullying/social exclusion*) and others were only about adult disclosure (e.g., *overreaction*). However, there was also an overlap of categories, for example, having trust in the confidant was mentioned as a reason for disclosure for both peers and adults. Yet, some corresponding categories differed slightly. Responses in the *help-seeking* category for adults more strongly expressed a competence to intervene attributed to the confidant (e.g., “His help and that he knows where to report what and how to deal with the situation”). In the *safety/fear* category, the concrete wish for protection was articulated more strongly for adults (e.g., “So that I feel safe and protected”).

#### Gender Differences

There were almost no gender differences in assessed reasons, except for two items regarding adult disclosure (*reporting of similar experiences by others*: *M*_girls_ = 3.02 (*SD* = 1.37), *M*_boys_ = 3.36 (*SD* = 1.29), *t*(376.2) = 2.56, *p* = 0.01, *d* = 0.26; *fear of punishment*: *M*_girls_ = 2.8 (*SD* = 1.49), *M*_boys_ = 2.5 (*SD* = 1.41), *t*(375.3) = −2.03, *p* = 0.04, *d* = 0.20).

Regarding open responses, female and male victims did not differ substantially in their willingness to provide further reasons, i.e., the proportions of those among female and male victims who gave further reasons were very similar for each open item. There were also hardly any gender differences in the derived categories, for example, approximately 10% of both male and female victims stated the wish to exchange as a reason for peer disclosure. There were only two exceptions: The proportion of female victims who mentioned help-seeking as a reason for adult disclosure was higher (girls: 16.1%, boys: 8.2%; χ²(1) = 4.73, *p* = 0.03, *h* = 0.24), while the proportion of male victims who reported further reasons for adult disclosure from the *Other* category was higher (girls: 3.0%, boys: 8.2%; χ²(1) = 4.30, *p* = 0.04, *h* = 0.23).

### Associations of Reasons With Actual Disclosure Behavior

#### Preliminary Descriptive Analyses

Associations of reasons with disclosure behavior were examined for the fourth objective. Before performing latent variable analyses, preliminary analyses compared disclosing and non-disclosing victims’ reasons for and against disclosure. Differences between disclosing and non-disclosing victims were evident for almost all reasons (see Table 5), with only two exceptions (*being asked* for peer disclosure, *blackmail/threats/interdictions by the perpetrator* for both). Reasons for disclosure were less strong, and reasons against disclosure were stronger among non-disclosing victims than disclosing victims. Differences were more pronounced in reasons regarding disclosure to adults, exhibiting medium effect sizes.

**Table 5:** Mean differences in reasons depending on disclosure behavior

Reason		Disclosing *M* (*SD*)	Non-disclosing *M* (*SD*)	Mean difference	|*d*|
For disclosure (peers)	Wish for victimization to end	3.67 (1.17)	3.35 (1.30)	−0.32*	0.27
	Wish for punishment of the perpetrator	3.35 (1.26)	3.05 (1.36)	−0.30*	0.24
	Wish for improvement of well-being	4.33 (0.85)	3.99 (0.99)	−0.34**	0.38
	Being asked	2.98 (1.14)	2.86 (1.23)	−0.12	–
	Reporting of similar experiences by others	3.76 (1.09)	3.17 (1.16)	−0.59***	0.54
Against disclosure (peers)	Embarrassment	3.22 (1.28)	3.61 (1.29)	0.39**	0.30
	Fear of disbelief	2.69 (1.36)	3.18 (1.35)	0.49**	0.36
	Fear of punishment	2.42 (1.36)	2.72 (1.33)	0.30*	0.22
	Blackmail/threats/interdictions by the perpetrator	2.10 (1.28)	2.24 (1.30)	0.14	–
For disclosure (adults)	Wish for victimization to end	4.26 (1.06)	3.54 (1.26)	−0.72***	0.62
	Wish for punishment of the perpetrator	3.85 (1.19)	3.13 (1.35)	−0.72***	0.57
	Wish for improvement of well-being	4.39 (0.83)	3.77 (1.21)	−0.62***	0.60
	Being asked	3.17 (1.25)	2.73 (1.24)	−0.44***	0.35
	Reporting of similar experiences by others	3.47 (1.34)	2.79 (1.27)	−0.68***	0.52
Against disclosure (adults)	Embarrassment	2.85 (1.43)	3.74 (1.25)	0.89***	0.66
	Fear of disbelief	2.38 (1.36)	2.98 (1.31)	0.60***	0.45
	Fear of punishment	2.27 (1.36)	3.16 (1.43)	0.89***	0.63
	Blackmail/threats/interdictions by the perpetrator	2.11 (1.25)	2.18 (1.26)	0.07	–

*** *p* < 0.001, ** *p* < 0.01, * *p* < 0.05. |*d*| = absolute Cohen’s *d*

#### Measurement Models (CFA)

Latent variable analyses were performed (a) to abstract from the item level (i.e., examining whether item groups shared an underlying latent construct), and (b) to contrast the relevance of reasons to disclosure to peers and adults (i.e., comparing standardized regression coefficients). First, measurement models of latent variables capturing cybergrooming victimization, as well as reasons for and against disclosure, had to be established with CFA (see Supplementary Material Fig. 3). The unidimensional measurement model of the MOGQ cybergrooming items had a good model fit in terms of SRMR and CFI. Still, the RMSEA slightly exceeded the acceptable fit threshold (see Table 6). The model fit of a unidimensional measurement model of reasons for disclosure was poor regarding both peers and adults (peers: χ²(5) = 73.17, RMSEA = 0.182, SRMR = 0.076, CFI = 0.851; adults: χ²(5) = 82.00, RMSEA = 0.200, SRMR = 0.079, CFI = 0.876). Therefore, a correlated factors model was examined with two factors of intraindividual reasons (*wish for victimization to end*, *wish for punishment of the perpetrator*, *wish for improvement of well-being*) and interindividual reasons (*being asked*, *reporting of similar experiences by others*). Again, SRMR and CFI indicated a good or acceptable model fit for peers and adults, while the RMSEA was relatively high. The unidimensional measurement model of reasons against disclosure indicated a good model fit for both peers and adults. These measurement models were considered appropriate despite higher RMSEA values, as in simple models with few degrees of freedom, as was the case here, the RMSEA can underestimate the model fit (Kenny et al., 2015). Further, SRMR and CFI consistently indicated acceptable to good model fit, and ω was acceptable to good in almost all cases (see Table 6). Factors of interindividual reasons for disclosure had the lowest ω, however, they had only two manifest indicators.

**Table 6:** Final measurement models

Model	χ^2^	df	RMSEA	SRMR	CFI	ω
Cybergrooming^a^	17.48	5	0.084	0.031	0.968	0.76
Reasons for disclosure (peers)^b^	31.34	4	0.127	0.044	0.942	0.79 (intra) 0.55 (inter)
Reasons for disclosure (adults)^b^	18.77	4	0.094	0.030	0.978	0.84 (intra) 0.67 (inter)
Reasons against disclosure (peers)^a^	2.41	2	0.025	0.014	0.999	0.75
Reasons against disclosure (adults)^a^	0.91	2	0.050	0.015	0.996	0.81

^a^Unidimensional model, ^b^Correlated factors model. ω = McDonald’s Omega

#### Structural Equation Models

**Fig. 1:**
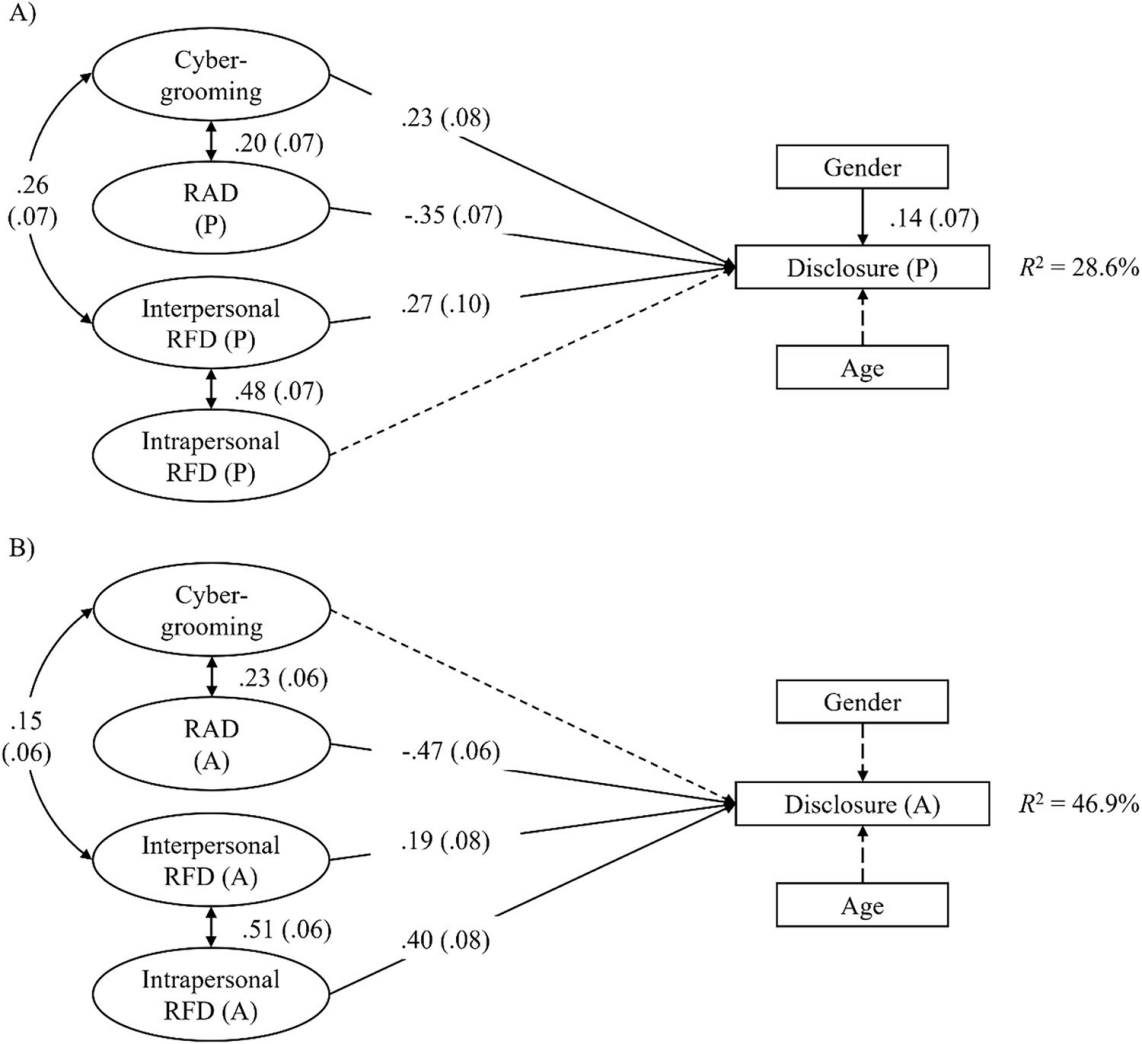
Prediction models for disclosure behavior. **A** Model for disclosure to peers, and **B** Model for disclosure to adults. RAD Reasons against disclosure, RFD Reasons for disclosure. All parameters are standardized. Non-significant regressions are displayed with dashed lines (*p* < 0.05). Figures in parentheses indicate standard errors. Loadings of manifest indicators on latent factors and non-significant correlations between latent predictors were omitted for clarity

Subsequently, associations between established latent variables and the manifest variable of disclosure behavior were examined in structural equation models. In separate models for disclosure to peers and adults, disclosure was regressed on cybergrooming, reasons, age, and gender. In both models, the CFI was problematic (peers: χ²(109) = 222.32, RMSEA = 0.05, SRMR = 0.06, CFI = 0.85; adults: χ²(109) = 254.53, RMSEA = 0.06, SRMR = 0.07, CFI = 0.81). After detailed inspection, one reason against disclosure (*blackmail/threats/interdictions by the perpetrator*) was removed from both models, as this improved model fit and non-disclosing and disclosing victims did not exhibit significant differences on this item in preliminary analyses. Both resulting models (see Fig. 1) demonstrated at least acceptable model fit (peers: χ²(94) = 163.23, RMSEA = 0.04, SRMR = 0.05, CFI = 0.90; adults: χ²(94) = 160.24, RMSEA = 0.04, SRMR = 0.05, CFI = 0.90). Disclosure to adults was most strongly predicted by reasons against disclosure (β = −0.47) and intrapersonal reasons for disclosure (β = 0.40), while the predictive power of interindividual reasons for disclosure was weaker (β = 0.19). Peer disclosure was also most strongly predicted by reasons against disclosure (β = −0.35). Compared to disclosure to adults, interindividual reasons for disclosure were more strongly predictive of peer disclosure (β = 0.27), while intrapersonal reasons for disclosure had no predictive power beyond this. The frequency of experienced cybergrooming situations only predicted peer disclosure (β = 0.23) but not disclosure to adults. There was a small effect of gender on peer disclosure (β = 0.14), reflecting the slightly higher rates of peer disclosure among girls. Despite the effects of cybergrooming and gender on peer disclosure, disclosure to adults was better explained (*R*^2^_peers_ = 28.6%, *R*^2^_adults_ = 46.9%). In both models, interindividual reasons for disclosure were positively correlated with intrapersonal reasons for disclosure and cybergrooming, and reasons against disclosure were also positively correlated with cybergrooming.

## Discussion

Disclosure processes and experiences following sexual victimization influence how victims cope with their victimization experiences. Identifying victims’ reasons for and against disclosure contributes to understanding and promoting disclosure processes. While there is emerging research on this in the context of cybergrooming victimization and related phenomena, reasons for disclosure and the role of different confidants in reasons for (non-)disclosure have scarcely been addressed. Therefore, the present study aimed to explore adolescents’ reasons for and against disclosure in the specific context of cybergrooming victimization. To pursue this goal, disclosure behavior and selected reasons for disclosure to peers and adults were quantitatively examined in 400 victims. In addition, participants’ open responses providing further reasons were categorized. Findings revealed (1) high disclosure rates, (2) several intra- and interpersonal reasons for and against disclosure, (3) differences in reasons depending on the intended confidant, and (4) associations between reasons and actual disclosure behavior.

### Cybergrooming Victimization Rate

With 44.1%, the prevalence rate of cybergrooming victimization was very high compared to previous studies. However, previously reported figures often indicated a 12-month prevalence, or less (e.g., Machimbarrena et al., 2018; Wachs et al., 2016), whereas a specific reference period was not used in the present study. Further, a low cut-off for classification as a cybergrooming victim was chosen, namely having experienced one of the cybergrooming situations at least once. This raises the question if some participants were incorrectly classified as cybergrooming victims. Instead, they may have experienced occasional events of sexual victimization (e.g., sexual solicitation or harassment) rather than the cybergrooming process. However, the exemplary chat sequences presented with each MOGQ item were designed to illustrate the conversational and longer-term nature of online contact with the perpetrator. Finally, results showed that most participants (83.3%) have experienced more than one cybergrooming situation, reducing the likelihood of their victimization experience being a single event.

### Disclosure Rates and Chosen Confidants

Concerning the first objective, the findings showed that, in total, 86% of victims disclosed to someone, indicating a higher disclosure rate than in previous studies, especially concerning disclosure to adults (Juusola et al., 2021; Villacampa & Gómez, 2017). Peer disclosure (73%) was still more prevalent than adult disclosure (55%), aligning with the current state of research on disclosure following sexual victimization (Manay & Collin-Vézina, 2021). It has been argued from a developmental psychology perspective that in adolescence, there is a shift in emotional attachment and perceived social support away from parents toward peers, leading to a preference for peer disclosure (Manay & Collin-Vézina, 2021). This tendency was reflected in responses of the *preferring peers over adults* category of further provided reasons (e.g., “Peers can understand it better and don’t make a fuss right away”, “The relationship with peers is better than with adults, especially as a teenager”, “One can talk to them better and possibly get advice if they have already experienced it”). The latter example also points to the importance of shared experience spaces as reflected in the *exchange of experiences* category. In this context, the digital component of online sexual victimization may contribute to the divergence between peer and adult disclosure rates following such victimization. Adolescents stated that they prefer to discuss digital sexual topics (i.e., sexting, pornography, online flirt/date) with their friends rather than their parents, as they perceived their parents to be less experienced with these newer forms of sexual expression (Widman et al., 2021). This aligns with research findings on victims of sexual solicitation preferring peer disclosure as they perceived their parents to be unfamiliar with experiences of online sexual abuse (Gemara et al., 2025). Still, regarding peers, the quality of relationships is central for the willingness to share and discuss secrets and personal information (e.g., Costello et al., 2024). While the high victimization rate as observed in the present study is worrisome, it is encouraging that most participants, particularly with respect to peers, appear to cultivate positive relationships, in which they are confident enough to disclose their cybergrooming experiences.

### Reasons For and Against Disclosure

Addressing the second objective, descriptive statistics of the reasons specified in closed-ended items indicated that, overall, selected reasons were considered in the disclosure process by a substantial proportion of victims. On average, agreement with reasons for disclosure was higher than with reasons against it. Recognizing that the selected reasons did not exhaustively represent all possible reasons, participants were asked to provide further reasons. They stated plenty of additional reasons, which were grouped into categories. Regarding both peer and adult disclosure, most categories of reasons for disclosure reflected characteristics of positive relationships, e.g., sharing and discussing experiences, anticipating help, support, or advice, and trusting the confidant. Two categories can further be associated with prosocial motivation, namely *warning/prevention* (i.e., the disclosure aimed to protect others from similar victimization experiences), and *encouragement* (i.e., the disclosure intended to encourage peers to disclose as well). Categories of reasons against peer disclosure were strongly characterized by fears of negative social consequences of disclosure, namely (a) being ridiculed, bullied, or excluded, (b) labeling effects (i.e., being seen as weird, stupid, or as a victim), and (c) spreading of their experiences and being topic of conversation. This illustrates that adolescents’ motivation to avoid social rejection strongly influences their decisions and behavior (Tomova et al., 2021). Categories of reasons against disclosure to adults likewise reflected negative reactions or consequences, namely *overreaction* and *lack of understanding*.

Evaluating these findings through a socio-ecological lens revealed that the reasons identified in the present study did not encompass the contextual domain. Instead, the identified reasons were intrapersonal (e.g., *wish for the victimization to end*, *embarrassment*) and interpersonal (e.g., *help-seeking*, *fear of disbelief*). In prior research, fear of labeling effects was considered a contextual reason and denoted, for instance, the fear of stigmas associated with rigid gender roles (e.g., being perceived as gay, weak, or “impure”; Collin-Vézina et al., 2015; Zinzow et al., 2022). However, the fear of labeling effects expressed by participants in the present study had strong interpersonal features (e.g., fear of being perceived as weird). Therefore, it could be regarded as an interpersonal reason. According to the socio-ecological model, contextual factors should also be considered to understand disclosure processes. For example, in previous research, CSA victims stated that they did not understand what was considered ‘normal’ or ‘not normal’ regarding sexuality during their childhood. They also lacked knowledge about sex, what constitutes inappropriate behavior or abuse, and where to find appropriate information (Collin-Vézina et al., 2015), resulting from a taboo on sexuality and acting as a barrier to disclosure. Likewise, in the context of cybergrooming victimization, adolescents often lack awareness of cybergrooming as an abusive, exploitative situation (Gámez-Guadix, Román, et al., 2021). Furthermore, perpetrators’ grooming strategies may include the justification and normalization of sexual behavior, such as sharing explicit material online (Gámez-Guadix & Mateos-Pérez, 2019). It can be hypothesized that an open discussion about sexuality and sexual victimization promotes the ability of victims to recognize the cybergrooming situation as such, or at least as unusual, which in turn could increase the likelihood of disclosure, especially to adults. Additionally, recalling that factors from different domains interact (Bronfenbrenner, 1979), it is conceivable that contextual factors influence intra- and interpersonal reasons. For example, societal attitudes associated with victim-blaming, such as attributing responsibility to victims based on clothing or online self-presentation (Flynn et al., 2023), may enhance self-blame and shame. However, this study was unable to identify these underlying contextual factors.

### Differences in Reasons For and Against Disclosure

#### Differences Depending on Confidants

Examining peer and adult disclosure in more detail for the third objective, several differences in reasons depending on confidants were observed. The *wish for the victimization to end* and the *wish for punishment of the perpetrator* were higher regarding disclosure to adults. This is in line with previous findings indicating that, in the context of CSA, adult disclosure is particularly motivated by the wish for the abuse to stop and for help in contacting authorities (Manay & Collin-Vézina, 2021). This anticipated competence to intervene attributed to adults was also evident in the *help-seeking* category. Although help-seeking was mentioned as a reason for both peer and adult disclosure, the intervening, action-oriented aspects were more salient for the latter (e.g., “Adults usually know best how to deal with such people. They also help you to solve the problem without necessarily having to contact the person online”). Further, *fear of punishment* was slightly higher for adult disclosure. In the context of online victimization, adolescents’ fear of Internet restrictions, parental interference in Internet usage behavior, and the confiscation of technical devices as punishment could be particularly pronounced, given how relevant and deeply rooted the Internet in general and social media in particular are in their social lives (e.g., Anderson et al., 2023). Victims have reported fears of this kind of punishment as a barrier to disclosure, for example, in the context of cyberbullying (Mishna et al., 2009) and online sexual solicitation (Gemara et al., 2025). In the present study, this fear was emphasized in some responses to open-ended items (e.g., “I would be afraid of no longer being allowed on the Internet”).

The victims’ *wish for improvement of well-being* was slightly higher regarding peer disclosure. This aligns with previous findings indicating that seeking emotional support is particularly relevant for peer disclosure (Manay & Collin-Vézina, 2021). Still, the respective effect size was very small in the current study, and the wish for well-being improvement was re-emphasized in open responses for both peer and adult disclosure. It can therefore be assumed that this is a strong reason for disclosure in general, regardless of the confidant. *Fear of disbelief* was also slightly higher for peer disclosure in the present study; however, it also had a very small effect size. By contrast, previous research on online sexual solicitation indicates greater fear of adverse reactions from adults than peers (Gemara et al., 2025). This could be linked, for example, to the fact that adults are perceived as less familiar with digital sexual topics and online sexual abuse than peers (Gemara et al., 2025; Widman et al., 2021), and peers are therefore more likely to believe the victim due to similar experiences they have already made. Greater fear of disbelief of peers, on the other hand, may stem rather from consequences of disbelief, for example, being seen as a liar or loss of trust on the part of the confidant, which could ultimately weaken the peer relationship. However, these are only hypothetical explanations that cannot be substantiated based on current research. Lastly, participants agreed more with *reporting of similar experiences by others* as a reason for peer disclosure than for adult disclosure, highlighting the role of peer influence in adolescents’ disclosure processes (McElvaney et al., 2014). It is conceivable that the disclosure of peers lowers the inhibition for one’s disclosure, or opens the possibility of jointly dealing with shared victimization experiences.

#### Gender Differences

With very few exceptions, the present results provided no evidence of gender differences in examined reasons and thus a similar pattern to previous quantitative research on gender differences in reasons for non-disclosure of CSA (Okur et al., 2020). However, this does not rule out the existence of more pronounced gender differences. In particular, the present study neglected contextual reasons in which gender differences might be found. For example, as previous research on sexual victimization suggests, men in particular are afraid of experiencing stigmatization and being perceived as a victim or labeled as “unmanly”, weak, or gay (Alaggia, 2005; Edwards et al., 2023). As adolescent development includes masculine role socialization (Rogers et al., 2021), it is conceivable that these fears already manifest in male adolescents.

### Associations of Reasons With Actual Disclosure Behavior

Concerning the fourth objective, preliminary analyses revealed differences in almost every reason according to disclosure behavior. Namely, disclosing victims agreed more strongly with reasons for disclosure and less strongly with reasons against disclosure than non-disclosing victims, except for *blackmail/threats/interdictions by the perpetrator*. This demonstrates that most selected reasons were, in fact, relevant to the actual disclosure decision. Accordingly, latent factors of reasons against disclosure and intraindividual and interindividual reasons for disclosure predicted disclosure behavior in structural equation models. Reasons against disclosure were the strongest predictor for actual disclosure behavior, both regarding peers and adults. Further, interpersonal and intrapersonal reasons for disclosure differed in their predictive power for peer and adult disclosure. Namely, intrapersonal reasons were particularly predictive of adult disclosure, while interpersonal reasons were more predictive of peer disclosure. This indicates that the examined reasons influence the disclosure decision to varying degrees depending on the confidants, which was also reflected in the fact that, overall, disclosure to adults was better explained than peer disclosure.

However, this should not imply that reasons against disclosure per se are more relevant for disclosure decisions. The main reason for this is that the latent factors reflected only a selection of reasons. In other words, the model did not cover all potential reasons for or against disclosure as predictors. Responses to the open-ended items revealed a variety of further reasons beyond the selected reasons that are relevant in the disclosure process. Further, building on prior research, it is conceivable that a lack of perceived seriousness of the situation in particular is a significant predictor of non-disclosure of cybergrooming victimization (Juusola et al., 2021; Villacampa & Gómez, 2017). However, a selection of reasons for the closed-ended items had to be made to design the questionnaire appropriately for the adolescent participants regarding completion time and cognitive load.

### Practical Implications

Two major practical implications of the present results shall be outlined. First, as emphasized by high peer disclosure rates, adolescents should be considered as key agents in the disclosure process and the progression and processing of victimization experiences. In a study investigating offline meetings stemming from cybergrooming processes, it was noted that confided peers could serve as a source of comfort but also as agents of safety to intervene (Chiu & Quayle, 2022). However, several issues may compromise peers’ perceived urgency or ability to intervene. There may be a lack of knowledge about cybergrooming as a phenomenon and a criminal offense, along with its recognition as an abusive situation (Gámez-Guadix, Román, et al., 2021; Wood & Wheatcroft, 2020). Some young people might even view cybergrooming situations as genuine relationships (Whittle et al., 2015). Therefore, adolescents should be educated about cybergrooming – not only to minimize the risk of becoming victims but also to prepare them for their role as confidants. Research indicates that school-based sex education is effective for preventing sexual abuse and promoting media literacy (Goldfarb & Lieberman, 2021). Keeping this in mind, incorporating cybergrooming into the sex education curriculum, as well as hosting additional events like workshops focusing on online sexual victimization, is a viable option. In this context, police officers, healthcare providers, and school social workers could – and should – be involved to provide young people with potential support services in the event of their victimization or a friend’s disclosure. However, such endeavors must respect adolescents’ evolving sexuality and desire for autonomy. As pointed out in the context of sexting, prevention and educational efforts should refrain from moralizing adolescents’ sexual behavior or even blaming them for engaging in such behavior (Wachs et al., 2021). Rather, both parent-initiated and school-based sex education should communicate sexual self-determination and consensual behavior in equal relationships.

Second, adequate reactions to disclosure can be derived from the results. The reactions should reflect the reasons for disclosure and contrast the reasons against disclosure. Participants strongly agreed that improving well-being was a reason for disclosure, and this was further emphasized in their open responses. The comments further indicated that participants expected or hoped for support from confidants through disclosure. This highlights the necessity of fostering a supportive environment after disclosure, as it primarily concerns victims’ well-being, safety, and needs. The findings revealed that victims face various fears regarding their confidants’ reactions, including disbelief, punishment, ridicule, and bullying. It is crucial to prevent such responses, as reactions to disclosure that are perceived as negative can harm the victim’s well-being beyond the victimization itself (Easton, 2019; McTavish et al., 2019). In addition, reactions based on well-meant intentions or lacking knowledge, sensibilization, or alternatives may not meet the victim’s needs. For example, although several victims wish for punishment of the perpetrator as evident in the present study, confidants’ endeavors to contact authorities to initiate the prosecution of the perpetrator must be undertaken with the victim’s will and state in mind, as they may not be emotionally prepared or willing to share their experience with formal agents like police officers (e.g., Ceelen et al., 2019). Alternatively, some adolescents might perceive it as an overreaction – a mentioned reason against disclosure – if parents immediately want to involve the police. Further, even if parents understood interference and restrictions on Internet use to protect their children from further victimization, young people could ultimately perceive it as a punishment (Priebe & Svedin, 2008). Consequently, potential confidants need to be sensitized and trained in appropriate reactions to disclosure. They should generally ensure that what is told is believed, offer emotional support, and show understanding. In peer disclosure in particular, positive peer relationships should be maintained by not treating the victim’s experiences as “gossip” or making fun of the victim or their experiences. Consequences of disclosure should not convey to the victim that their social standing or network is at risk. Further, for adult confidants in particular, offering tangible and targeted help and intervention options and avoiding punishment, especially about Internet use, are highly relevant.

### Limitations

First, the generalization of the present findings is limited due to the sample’s composition, as it was well-educated, gender minorities were not included, and only adolescents were surveyed. Gender minorities were neglected, as the case number of victims with a gender other than male and female was very low, and analyses, therefore, incorporated gender only as a binary variable. However, previous research suggests that there are reasons against disclosure of sexual victimization that are unique to sexual and gender minorities, such as fear of shedding negative light on the LGBTQ+ community or being “outed” (Edwards et al., 2023). Further, disclosure processes may vary between children and adolescents. For instance, disclosure to parents and involuntary and prompted disclosures were more prevalent in younger children than adolescents (Manay & Collin-Vézina, 2021). Disclosure is particularly relevant for younger children, as they may be confronted with sexual content for the first time when experiencing cybergrooming victimization, which they might be unable to deal with on their own. Future studies should include more diverse samples regarding age, gender, and education.

Second, in contrast to most previous studies on cybergrooming victimization, a reference period was not specified. All participants who had ever experienced cybergrooming and were willing or able to report this were to be identified to gain as much information as possible. This also allowed to include victims who disclosed with a greater delay. However, also considering potential memory deficits after adverse life events (Meyer & Benoit, 2022; Zoellner et al., 2019), the recall bias (Kihlstrom et al., 2000) could be particularly pronounced in victims whose victimization experiences occurred several years ago. This could have influenced adolescents’ retrospective self-reports on victimization and disclosure experiences, for example about the frequency of cybergrooming experiences or the relative relevance of specific reasons at the time of the disclosure process.

Third, the dichotomous measurement of disclosure used here does not reflect disclosure well as a process but rather as a single event, and, thus, does not allow for information on changes in decisions, delays in disclosure, or interactions between peer and adult disclosure. The potential preceding and preparing role of peer disclosure for disclosure to adults was evident in open responses (“As it happens to many people our age, it’s good to have someone to talk to before you talk to your parents, for example”, “Maybe you hope to get somehow the parents involved through a friend”). Gaining insights into dynamics within the process would considerably enhance the understanding of disclosure processes. Therefore, future studies should investigate, e.g., (a) which confidant was chosen for the first disclosure, (b) the sequence of disclosures to different confidants, (c) the influence of reactions to disclosure on further disclosure, and (d) the delay between victimization and the first disclosure, and between disclosure to different confidants. In this context, longitudinal studies and innovative data sampling strategies (e.g., ecological momentary assessment) would strongly enrich the current body of research, firstly by making the measurement of the process more reliable, as victims would not have to recall and report the entire process at once, and secondly by identifying temporal relationships, e.g., between first confidants’ reactions and following disclosure behavior.

Finally, the present study does not provide information on the role of contextual reasons and formal recipients in disclosure processes. Participants had the opportunity to indicate adult formal confidants and contextual reasons in open-ended items. However, it is conceivable that they were primarily thinking of (1) disclosure situations with adults from their close environment and not, for example, police reporting situations, and (2) intra- and interpersonal reasons rather than analyzing the influence of societal structures and norms on their disclosure behavior. Future studies could explicitly ask victims whether, why, and when they have disclosed to formal recipients. Since contextual reasons may require greater capacity for abstraction and are therefore more difficult to assess directly in youth surveys, multi-level analyses represent a compelling alternative for examining contextual factors, with adolescents nested in, for instance, families, schools, communities, and/or countries. For example, when analyzing disclosure behavior of victims nested in different schools, several predictors of disclosure behavior at the school level are eligible for analysis. This could reflect the degree to which sex education is integrated into the curriculum and implemented by teachers, as well as the extent to which schools invest in preventive measures against sexual victimization and organize additional project weeks or informative events that facilitate open discussions on the topic and highlight available support services.

## Conclusion

Cybergrooming represents a prevalent type of online sexual victimization during adolescence. Disclosure has the potential to positively influence the course of victimization experiences and victims’ well-being. Nevertheless, despite its importance, little is known about why adolescents disclose or withhold experiences of cybergrooming victimization. This study, therefore, aimed to provide insights into adolescents’ (non-)disclosure behavior and its underlying reasons, considering peers and adults as potential confidants. Based on self-report data of cybergrooming victims, findings indicated high disclosure rates, especially with regard to peers (total: 86%, peers: 73%, adults: 55%). A variety of intra- and interpersonal reasons were identified that are potentially involved in disclosure processes, for instance, the wish for improvement of well-being, help-seeking, and exchange of experiences as reasons for disclosure, and embarrassment as well as fear of negative reactions and consequences such as disbelief, punishment, bullying, and social exclusion as reasons against disclosure. Findings further suggest that peer and adult disclosure differ in (strength of) underlying reasons, and that selected reasons are linked to the actual disclosure behavior. Future research is recommended on processual dynamics of disclosure of cybergrooming experiences as well as contextual reasons for and against such disclosure. Still, the present findings highlight the key role of peers in the disclosure process and inform how confidants should react to disclosure to promote positive disclosure experiences for victims.

## Supplementary information

Supplementary Material

## Data Availability

Data is provided in an OSF online repository (https://osf.io/aqd5k/files/osfstorage).

## Code Availability

R scripts and, codebook are provided in an OSF online repository (https://osf.io/aqd5k/files/osfstorage).

## References

[CR1] Alaggia, R. (2005). Disclosing the trauma of child sexual abuse: A gender analysis. *Journal of Loss and Trauma*, *10*(5), 453–470. 10.1080/15325020500193895.

[CR2] Alaggia, R., Collin-Vézina, D., & Lateef, R. (2019). Facilitators and barriers to child sexual abuse (CSA) disclosures: A research update (2000–2016). *Trauma, Violence & Abuse*, *20*(2), 260–283. 10.1177/1524838017697312. 10.1177/1524838017697312 PMC6429637 29333973

[CR3] Anderson, M., Faverio, M., & Gottfried, J. (2023). *Teens, social media and technology 2023*. Pew Research Center. https://abfe.issuelab.org/resources/43096/43096.pdf

[CR4] Beauducel, A., & Herzberg, P. Y. (2006). On the performance of maximum likelihood versus means and variance adjusted weighted least squares estimation in CFA. *Structural Equation Modeling: A Multidisciplinary Journal*, *13*(2), 186–203. 10.1207/s15328007sem1302_2.

[CR5] Bentler, P. M. (1990). Comparative fit indexes in structural models. *Psychological Bulletin*, *107*(2), 238–246. 10.1037/0033-2909.107.2.238. 2320703 10.1037/0033-2909.107.2.238

[CR6] Bergmann, M. C., & Baier, D. (2016). Erfahrungen von jugendlichen mit cybergrooming: Schülerbefragung - jugenddelinquenz. *RPsych - Rechtspsychologie*, *2*(2), 172–189. 10.5771/2365-1083-2016-2-172.

[CR7] Brattfjell, M. L., & Flåm, A. M. (2019). They were the ones that saw me and listened.” From child sexual abuse to disclosure: Adults’ recalls of the process towards final disclosure. *Child Abuse & Neglect*, *89*, 225–236. 10.1016/j.chiabu.2018.11.022. 30639125 10.1016/j.chiabu.2018.11.022

[CR8] Bronfenbrenner, U. (1979). *The ecology of human development: Experiments by nature and design*. Cambridge, MA: Harvard University Press.

[CR9] Browne, M. W., & Cudeck, R. (1992). Alternative ways of assessing model fit. *Sociological Methods & Research*, *21*(2), 230–258. 10.1177/0049124192021002005.

[CR10] Calvete, E., Fernández-González, L., González-Cabrera, J., Machimbarrena, J. M., & Orue, I. (2020). Internet-risk classes of adolescents, dispositional mindfulness and health-related quality of life: A mediational model. *Cyberpsychology, Behavior and Social Networking*, *23*(8), 533–540. 10.1089/cyber.2019.0705. 32391724 10.1089/cyber.2019.0705

[CR11] Castellanos, M., Wettstein, A., Wachs, S., Kansok-Dusche, J., Ballaschk, C., Krause, N., & Bilz, L. (2023). Hate speech in adolescents: A binational study on prevalence and demographic differences. *Frontiers in Education*, *8*, 1–14. 10.3389/feduc.2023.1076249.

[CR12] Ceelen, M., Dorn, T., van Huis, F. S., & Reijnders, U. J. L. (2019). Characteristics and post-decision attitudes of non-reporting sexual violence victims. *Journal of Interpersonal Violence*, *34*(9), 1961–1977. 10.1177/0886260516658756. 27402581 10.1177/0886260516658756

[CR13] Chiu, J., & Quayle, E. (2022). Understanding online grooming: An interpretative phenomenological analysis of adolescents’ offline meetings with adult perpetrators. *Child Abuse & Neglect*, *128*, 105600. 10.1016/j.chiabu.2022.105600. 35338948 10.1016/j.chiabu.2022.105600

[CR14] Collin-Vézina, D., de La Sablonnière-Griffin, M., Palmer, A. M., & Milne, L. (2015). A preliminary mapping of individual, relational, and social factors that impede disclosure of childhood sexual abuse. *Child Abuse & Neglect*, *43*, 123–134. 10.1016/j.chiabu.2015.03.010. 25846196 10.1016/j.chiabu.2015.03.010

[CR15] Costello, M. A., Pettit, C., Hellwig, A. F., Hunt, G. L., Bailey, N. A., & Allen, J. P. (2024). Adolescent social learning within supportive friendships: Self-disclosure and relationship quality from adolescence to adulthood. *Journal of Research on Adolescence*, *34*(3), 805–817. 10.1111/jora.12947. 38650089 10.1111/jora.12947 PMC11349471

[CR16] Easton, S. D. (2019). Childhood disclosure of sexual abuse and mental health outcomes in adulthood: Assessing merits of early disclosure and discussion. *Child Abuse & Neglect*, *93*, 208–214. 10.1016/j.chiabu.2019.04.005. 31121521 10.1016/j.chiabu.2019.04.005 PMC6545143

[CR17] Easton, S. D., Saltzman, L. Y., & Willis, D. G. (2014). “Would you tell under circumstances like that?”: Barriers to disclosure of child sexual abuse for men. *Psychology of Men & Masculinities*, *15*(4), 460–469. 10.1037/a0034223.

[CR18] Edwards, K. M., Waterman, E. A., Ullman, S. E., Rodriguez, L. M., Dardis, C. M., & Dworkin, E. R. (2022). A pilot evaluation of an intervention to improve social reactions to sexual and partner violence disclosures. *Journal of Interpersonal Violence*, *37*(5-6), 2510–2534. 10.1177/0886260520934437. 32646275 10.1177/0886260520934437 PMC7796907

[CR19] Edwards, K. M., Mauer, V. A., Huff, M., Farquhar-Leicester, A., Sutton, T. E., & Ullman, S. E. (2023). Disclosure of sexual assault among sexual and gender minorities: A systematic literature review. *Trauma, Violence & Abuse*, *24*(3), 1608–1623. 10.1177/15248380211073842. 10.1177/15248380211073842 35403506

[CR20] Eleuteri, S., Saladino, V., & Verrastro, V. (2017). Identity, relationships, sexuality, and risky behaviors of adolescents in the context of social media. *Sexual and Relationship Therapy*, *32*(3-4), 354–365. 10.1080/14681994.2017.1397953.

[CR21] Feierabend, S., Rathgeb, T., Kheredmand, H., & Glöckler, S. (2022). *JIM-studie 2022. jugend, information, medien. basisuntersuchung zum medienumgang 12- bis 19-jähriger in Deutschland*. https://www.lfk.de/fileadmin/user_upload/jim-studie-2022.pdf

[CR22] Flynn, A., Cama, E., Powell, A., & Scott, A. J. (2023). Victim-blaming and image-based sexual abuse. *Journal of Criminology*, *56*(1), 7–25. 10.1177/26338076221135327.

[CR23] Gámez-Guadix, M., & Mateos-Pérez, E. (2019). Longitudinal and reciprocal relationships between sexting, online sexual solicitations, and cyberbullying among minors. *Computers in Human Behavior*, *94*, 70–76. 10.1016/j.chb.2019.01.004.

[CR24] Gámez-Guadix, M., De Santisteban, P., & Alcazar, M. A. (2018). The construction and psychometric properties of the questionnaire for online sexual solicitation and interaction of minors with adults. *Sexual Abuse*, *30*(8), 975–991. 10.1177/1079063217724766. 28821214 10.1177/1079063217724766

[CR25] Gámez-Guadix, M., De Santisteban, P., Wachs, S., & Wright, M. (2021). Unraveling cyber sexual abuse of minors: Psychometrics properties of the multidimensional online grooming questionnaire and prevalence by sex and age. *Child Abuse & Neglect*, *120*, 105250. 10.1016/j.chiabu.2021.105250. 34399230 10.1016/j.chiabu.2021.105250

[CR26] Gámez-Guadix, M., Román, F. J., Mateos, E., & De Santisteban, P. (2021). Creencias erróneas sobre el abuso sexual online de menores (“child grooming”) y evaluación de un programa de prevención. *Behavioral Psychology/Psicología Conductual*, *29*(2), 283–296. 10.51668/bp.8321204s.

[CR27] Gámez-Guadix, M., Mateos-Pérez, E., Alcázar, M. A., Martínez-Bacaicoa, J., & Wachs, S. (2023). Stability of the online grooming victimization of minors: Prevalence and association with shame, guilt, and mental health outcomes over one year. *Journal of Adolescence*, *95*(8), 1715–1724. 10.1002/jad.12240. 37661357 10.1002/jad.12240

[CR28] Gemara, N., Mishna, F., & Katz, C. (2025). ‘If my parents find out, I will not see my phone anymore’: Who do children choose to disclose online sexual solicitation to? *Child & Family Social Work*, *30*(1), 4–14. 10.1111/cfs.13069.

[CR30] Giordano, P. C. (2003). Relationships in adolescence. *Annual Review of Sociology*, *29*(1), 257–281. 10.1146/annurev.soc.29.010202.100047.

[CR31] Goldfarb, E. S., & Lieberman, L. D. (2021). Three decades of research: The case for comprehensive sex education. *The Journal of Adolescent Health*, *68*(1), 13–27. 10.1016/j.jadohealth.2020.07.036. 33059958 10.1016/j.jadohealth.2020.07.036

[CR32] Haddock, A., Ward, N., Yu, R., & O’Dea, N. (2022). Positive effects of digital technology use by adolescents: A scoping review of the literature. *International Journal of Environmental Research and Public Health*, *19*(21), 14009. 10.3390/ijerph192114009. 10.3390/ijerph192114009 PMC9658971 36360887

[CR33] Hu, L., & Bentler, P. M. (1999). Cutoff criteria for fit indexes in covariance structure analysis: Conventional criteria versus new alternatives. *Structural Equation Modeling: A Multidisciplinary Journal*, *6*(1), 1–55. 10.1080/10705519909540118.

[CR34] Jackson, M. A., Valentine, S. E., Woodward, E. N., & Pantalone, D. W. (2017). Secondary victimization of sexual minority men following disclosure of sexual assault: “Victimizing me all over again…”. *Sexuality Research and Social Policy*, *14*(3), 275–288. 10.1007/s13178-016-0249-6.

[CR35] Jacques-Tiura, A. J., Tkatch, R., Abbey, A., & Wegner, R. (2010). Disclosure of sexual assault: Characteristics and implications for posttraumatic stress symptoms among African American and caucasian survivors. *Journal of Trauma & Dissociation*, *11*(2), 174–192. 10.1080/15299730903502938. 20373205 10.1080/15299730903502938 PMC2862213

[CR36] Juusola, A., Simola, T., Tasa, J., Karhu, E., & Sillfors, P. (2021). *Grooming in the eyes of a child - A report on the experiences of children on online grooming*. https://pelastakaalapset.s3.eu-west-1.amazonaws.com/main/2021/08/03151159/grooming_in_the_eyes_of_a_child_2021.pdf

[CR37] Kalkbrenner, M. T. (2023). Alpha, omega, and H internal consistency reliability estimates: Reviewing these options and when to use them. *Counseling Outcome Research and Evaluation*, *14*(1), 77–88. 10.1080/21501378.2021.1940118.

[CR38] Katz, C. (2013). Internet-related child sexual abuse: What children tell us in their testimonies. *Children and Youth Services Review*, *35*(9), 1536–1542. 10.1016/j.childyouth.2013.06.006.

[CR39] Katz, C., Piller, S., Glucklich, T., & Matty, D. E. (2021). “Stop waking the dead”: Internet child sexual abuse and perspectives on its disclosure. *Journal of Interpersonal Violence*, *36*(9-10), NP5084–NP5104. 10.1177/0886260518796526. 30160592 10.1177/0886260518796526

[CR40] Kellogg, N. D., & Huston, R. L. (1995). Unwanted sexual experiences in adolescents: Patterns of disclosure. *Clinical Pediatrics*, *34*(6), 306–312. 10.1177/000992289503400603. 7656510 10.1177/000992289503400603

[CR41] Kenny, D., Kaniskan, B., & Mccoach, D. B. (2015). The performance of RMSEA in models with small degrees of freedom. *Sociological Methods & Research*, *44*(3), 486–507. 10.1177/0049124114543236.

[CR42] Kihlstrom, J. F., Eich, E., Sandbrand, D., & Tobias, B. A. (2000). Emotion and memory: Implications for self-report. In A. A. Stone, J. S. Turkkan, C. A. Bachrach, J. B. Jobe, H. S. Kurtzman, & V. S. Cain (Eds.), *The science of self-report: Implications for research and practice* (pp. 81–99). Mahwah: Lawrence Erlbaum Associates Publishers.

[CR43] Lahtinen, H. ‑M., Laitila, A., Korkman, J., & Ellonen, N. (2018). Children’s disclosures of sexual abuse in a population-based sample. *Child Abuse & Neglect*, *76*, 84–94. 10.1016/j.chiabu.2017.10.011. 29096161 10.1016/j.chiabu.2017.10.011

[CR44] Landis, J. R., & Koch, G. G. (1977). The measurement of observer agreement for categorical data. *Biometrics*, *33*(1), 159. 10.2307/2529310. 843571

[CR45] Lemaigre, C., Taylor, E. P., & Gittoes, C. (2017). Barriers and facilitators to disclosing sexual abuse in childhood and adolescence: A systematic review. *Child Abuse & Neglect*, *70*, 39–52. 10.1016/j.chiabu.2017.05.009. 28551460 10.1016/j.chiabu.2017.05.009

[CR46] Li, C., Wang, P., Martin-Moratinos, M., Bella‑Fernández, M., & Blasco‑Fontecilla, H. (2024). Traditional bullying and cyberbullying in the digital age and its associated mental health problems in children and adolescents: A meta-analysis. *European Child & Adolescent Psychiatry*, *33*, 2895–2909. 10.1007/s00787-022-02128-x. 36585978 10.1007/s00787-022-02128-x PMC11424704

[CR47] Machimbarrena, J. M., Calvete, E., Fernández-González, L., Álvarez-Bardón, A., Álvarez-Fernández, L., & González-Cabrera, J. (2018). Internet risks: An overview of victimization in cyberbullying, cyber dating abuse, sexting, online grooming and problematic internet use. *International Journal of Environmental Research and Public Health*, *15*(11), Article 2471. 10.3390/ijerph15112471. 30400659 10.3390/ijerph15112471 PMC6267617

[CR48] Manay, N., & Collin-Vézina, D. (2021). Recipients of children’s and adolescents’ disclosures of childhood sexual abuse: A systematic review. *Child Abuse & Neglect*, *116*(Pt 1), Article 104192. 10.1016/j.chiabu.2019.104192. 31564382 10.1016/j.chiabu.2019.104192

[CR49] Mapes, A. R., & Cavell, T. A. (2021). Perceived barriers, relationship quality, and informal mentors: Adolescents’ preference for disclosing about dating violence. *Journal of Community Psychology*, *49*(7), 2719–2737. 10.1002/jcop.22666. 34260746 10.1002/jcop.22666

[CR50] Maschke, S., & Stecher, L. (2021). *»Speak!« Die studie. Sexualisierte gewalt in der erfahrung jugendlicher. Erweiterungsstudie berufliche schulen. Kurzbericht*. https://www.uni-marburg.de/de/fb21/erzwinst/arbeitsbereiche/aew/forschung/speak/kurzbericht-speak-berufliche-schulen_maschke-stecher.pdf

[CR51] McDonald, R. P. (1999). *Test theory: A unified treatment* (1st ed.). New York: Psychology Press. 10.4324/9781410601087.

[CR52] McElvaney, R., Greene, S., & Hogan, D. (2014). To tell or not to tell? Factors influencing young people’s informal disclosures of child sexual abuse. *Journal of Interpersonal Violence*, *29*(5), 928–947. 10.1177/0886260513506281. 24288188 10.1177/0886260513506281

[CR53] McTavish, J. R., Sverdlichenko, I., MacMillan, H. L., & Wekerle, C. (2019). Child sexual abuse, disclosure and PTSD: A systematic and critical review. *Child Abuse & Neglect*, *92*, 196–208. 10.1016/j.chiabu.2019.04.006. 30999168 10.1016/j.chiabu.2019.04.006

[CR54] Mennicke, A., Coates, C. A., Jules, B., & Langhinrichsen-Rohling, J. (2022). Who do they tell? College students’ formal and informal disclosure of sexual violence, sexual harassment, stalking, and dating violence by gender, sexual identity, and race. *Journal of Interpersonal Violence*, *37*(21-22), NP20092–NP20119. 10.1177/08862605211050107. 34798795 10.1177/08862605211050107

[CR55] Meyer, A. ‑K., & Benoit, R. G. (2022). Suppression weakens unwanted memories via a sustained reduction of neural reactivation. *eLife*, *11*, Article e71309. 10.7554/eLife.71309. 35352679 10.7554/eLife.71309 PMC8967383

[CR56] Mishna, F., Saini, M., & Solomon, S. (2009). Ongoing and online: Children and youth’s perceptions of cyber bullying. *Children and Youth Services Review*, *31*(12), 1222–1228. 10.1016/j.childyouth.2009.05.004.

[CR57] Moosburner, M., Weber, C., Kuban, T., Wachs, S., Schmidt, A. F., Etzler, S., & Rettenberger, M. (2025). Understanding cybergrooming: A systematic review of perpetrator characteristics, strategies, and types. *Trauma, Violence & Abuse*. 10.1177/15248380251316223 10.1177/15248380251316223 39963981

[CR58] Von Moy, Y. (2012). *Disclosure und soziale reaktionen bei betroffenen von missbrauch durch vertreter der katholischen kirche* [Diploma thesis, University of Vienna]. PHAIDRA. https://phaidra.univie.ac.at/o:1290663

[CR59] Okur, P., Van der Knaap, L. M., & Bogaerts, S. (2020). A quantitative study on gender differences in disclosing child sexual abuse and reasons for nondisclosure. *Journal of Interpersonal Violence*, *35*(23-24), 5255–5275. 10.1177/0886260517720732. 29294841 10.1177/0886260517720732

[CR60] Patel, U., & Roesch, R. (2022). The prevalence of technology-facilitated sexual violence: A meta-analysis and systematic review. *Trauma, Violence & Abuse*, *23*(2), 428–443. 10.1177/1524838020958057. 10.1177/1524838020958057 32930064

[CR61] Priebe, G., & Svedin, C. G. (2008). Child sexual abuse is largely hidden from the adult society: An epidemiological study of adolescents’ disclosures. *Child Abuse & Neglect*, *32*(12), 1095–1108. 10.1016/j.chiabu.2008.04.001. 19038448 10.1016/j.chiabu.2008.04.001

[CR62] Priebe, G., Mitchell, K. J., & Finkelhor, D. (2013). To tell or not to tell? Youth’s responses to unwanted internet experiences. *Cyberpsychology: Journal of Psychosocial Research on Cyberspace*, *7*(1), Article 6. 10.5817/CP2013-1-6.

[CR63] R Core Team. (2024). *R: A language and environment for statistical computing* (Version 4.4.2) [Computer software]. R Foundation for Statistical Computing. https://www.R-project.org/.

[CR64] Rau, T., Ohlert, J., Fegert, J. M., & Allroggen, M. (2016). Disclosure von jugendlichen in jugendhilfeeinrichtungen und internaten nach sexueller gewalterfahrung. *Praxis der Kinderpsychologie und Kinderpsychiatrie*, *65*(9), 638–654. 10.13109/prkk.2016.65.9.638. 27819617 10.13109/prkk.2016.65.9.638

[CR65] Ray, A., & Henry, N. (2025). Sextortion: A scoping review. *Trauma, Violence & Abuse*, *26*(1), 138–155. 10.1177/15248380241277271. 10.1177/15248380241277271 PMC11558931 39323232

[CR66] Ringenberg, T. R., Seigfried-Spellar, K. C., Rayz, J. M., & Rogers, M. K. (2022). A scoping review of child grooming strategies: Pre- and post-internet. *Child Abuse & Neglect*, *123*, Article 105392. 10.1016/j.chiabu.2021.105392. 34801848 10.1016/j.chiabu.2021.105392

[CR67] Rogers, A. A., Nielson, M. G., & Santos, C. E. (2021). Manning up while growing up: A developmental-contextual perspective on masculine gender-role socialization in adolescence. *Psychology of Men & Masculinities*, *22*(2), 354–364. 10.1037/men0000296.

[CR68] Rosseel, Y. (2012). lavaan: An R package for structural equation modeling. *Journal of Statistical Software*, *48*(2), 1–36. 10.18637/jss.v048.i02.

[CR69] Schaeffer, P., Leventhal, J. M., & Asnes, A. G. (2011). Children’s disclosures of sexual abuse: Learning from direct inquiry. *Child Abuse & Neglect*, *35*(5), 343–352. 10.1016/j.chiabu.2011.01.014. 21620161 10.1016/j.chiabu.2011.01.014

[CR70] Schittenhelm, C., Kops, M., Moosburner, M., Fischer, S. M., & Wachs, S. (2024). Cybergrooming victimization among young people: A systematic review of prevalence rates, risk factors, and outcomes. *Adolescent Research Review*. Advance online publication. 10.1007/s40894-024-00248-w

[CR71] Senekal, J. S., Ruth Groenewald, G., Wolfaardt, L., Jansen, C., & Williams, K. (2023). Social media and adolescent psychosocial development: A systematic review. *South African Journal of Psychology*, *53*(2), 157–171. 10.1177/00812463221119302.

[CR72] Srivastava, A., Rusow, J., Schrager, S. M., Stephenson, R., & Goldbach, J. T. (2022). Digital sexual violence and suicide risk in a national sample of sexual minority adolescents. *Journal of Interpersonal Violence*, *38*(3-4), 4443–4458. 10.1177/08862605221116317. 35942940 10.1177/08862605221116317 PMC9850373

[CR73] Tomova, L., Andrews, J. L., & Blakemore, S. ‑J. (2021). The importance of belonging and the avoidance of social risk taking in adolescence. *Developmental Review*, *61*, Article 100981. 10.1016/j.dr.2021.100981.

[CR29] Van Gijn-Grosvenor, E. L., & Lamb, M. E. (2021). Online groomer typology scheme. *Psychology, Crime & Law*, *27*(10), 973–987. 10.1080/1068316X.2021.1876048.

[CR74] Villacampa, C., & Gómez, M. J. (2017). Online child sexual grooming: Empirical findings on victimisation and perspectives on legal requirements. *International Review of Victimology*, *23*(2), 105–121. 10.1177/0269758016682585.

[CR75] Wachs, S., Wolf, K. D., & Pan, C. ‑C. (2012). Cybergrooming: Risk factors, coping strategies and associations with cyberbullying. *Psicothema*, *24*(4), 628–633. https://reunido.uniovi.es/index.php/pst/article/view/9714 23079362

[CR76] Wachs, S., Wright, M. F., Gámez-Guadix, M., & Döring, N. (2021). How are consensual, non-consensual, and pressured sexting linked to depression and self-harm? The moderating effects of demographic variables. *International Journal of Environmental Research and Public Health*, *18*(5), Article 2597. 10.3390/ijerph18052597. 33807667 10.3390/ijerph18052597 PMC7967514

[CR77] Wachs, S., Jiskrova, G. K., Vazsonyi, A. T., Wolf, K. D., & Junger, M. (2016). A cross-national study of direct and indirect effects of cyberbullying on cybergrooming victimization via self-esteem. *Educational Psychology (Psicología educativa)*, *22*(1), 61–70. 10.1016/j.pse.2016.01.002.

[CR78] Webster, S., Davidson, J., Bifulco, A., Gottschalk, P., Caretti, V., Pham, T., Grove-Hills, J., Turley, C., Tompkins, C., Ciulla, S., Milazzo, V., Schimmenti, A., & Craparo, G. (2012). *European online grooming project - final report*. www.researchgate.net/publication/257941820_European_Online_Grooming_Project_-_Final_Report

[CR79] Whittle, H., Hamilton-Giachritsis, C., & Beech, A. (2013). Victims’ voices: The impact of online grooming and sexual abuse. *Universal Journal of Psychology*, *1*(2), 59–71. 10.13189/ujp.2013.010206.

[CR80] Whittle, H., Hamilton-Giachritsis, C. E., & Beech, A. R. (2015). A comparison of victim and offender perspectives of grooming and sexual abuse. *Deviant Behavior*, *36*(7), 539–564. 10.1080/01639625.2014.944074.

[CR81] Widman, L., Javidi, H., Maheux, A. J., Evans, R., Nesi, J., & Choukas-Bradley, S. (2021). Sexual communication in the digital age: Adolescent sexual communication with parents and friends about sexting, pornography, and starting relationships online. *Sexuality & Culture*, *25*(6), 2092–2109. 10.1007/s12119-021-09866-1.

[CR82] Wolak, J., Finkelhor, D., Walsh, W., & Treitman, L. (2018). Sextortion of minors: Characteristics and dynamics. *Journal of Adolescent Health*, *62*(1), 72–79. 10.1016/j.jadohealth.2017.08.014. 10.1016/j.jadohealth.2017.08.014 29055647

[CR83] Wood, A. C., & Wheatcroft, J. M. (2020). Young adult perceptions of internet communications and the grooming concept. *SAGE Open*, *10*(1), Article 2158244020914573. 10.1177/2158244020914573.

[CR84] Wright, M. F., & Wachs, S. (2024). Longitudinal associations between different types of sexting, adolescent mental health, and sexual risk behaviors: Moderating effects of gender, ethnicity, disability status, and sexual minority status. *Archives of Sexual Behavior*, *53*, 1115–1128. 10.1007/s10508-023-02764-7. 38216785 10.1007/s10508-023-02764-7

[CR85] Zinzow, H. M., Littleton, H., Muscari, E., & Sall, K. (2022). Barriers to formal help-seeking following sexual violence: Review from within an ecological systems framework. *Victims & Offenders*, *17*(6), 893–918. 10.1080/15564886.2021.1978023.

[CR86] Zoellner, L. A., Sheikh, I. S., Walker, R. W., Rosencrans, P., Garcia, N. M., Marks, E. H., Ojalehto, H. J., & Bedard-Gilligan, M. A. (2019). Sexual assault and memory. In W. T. O’Donohue, & P. A. Schewe (Eds.), *Handbook of Sexual Assault and Sexual Assault Prevention* (pp. 337–352). Cham: Springer. 10.1007/978-3-030-23645-8_20.

