# Supplementary material for: Why They Speak Up (or Don’t): Reasons For and Against Cybergrooming Disclosure Among Adolescent Victims
Source: J Youth Adolesc. 2025 May 12;54(9):2269–87. doi: 10.1007/s10964-025-02192-x (PMC12420724; doi:10.1007/s10964-025-02192-x)
Supplement: Supplementary file 1 — Supplementary Material [file 10964_2025_2192_MOESM1_ESM.docx]

# Supplementary Materials

Article title: Why They Speak Up (or Don’t): Reasons For and Against Cybergrooming Disclosure Among Adolescent Victims

Authors: Catherine Schittenhelm, Christine Weber, Maxime Kops, Sebastian Wachs Corresponding author’s contact information: catherine.schittenhelm@uni-muenster.de

Journal: Journal of Youth and Adolescence

**SM Figure 1**

*Data Cleaning Process*


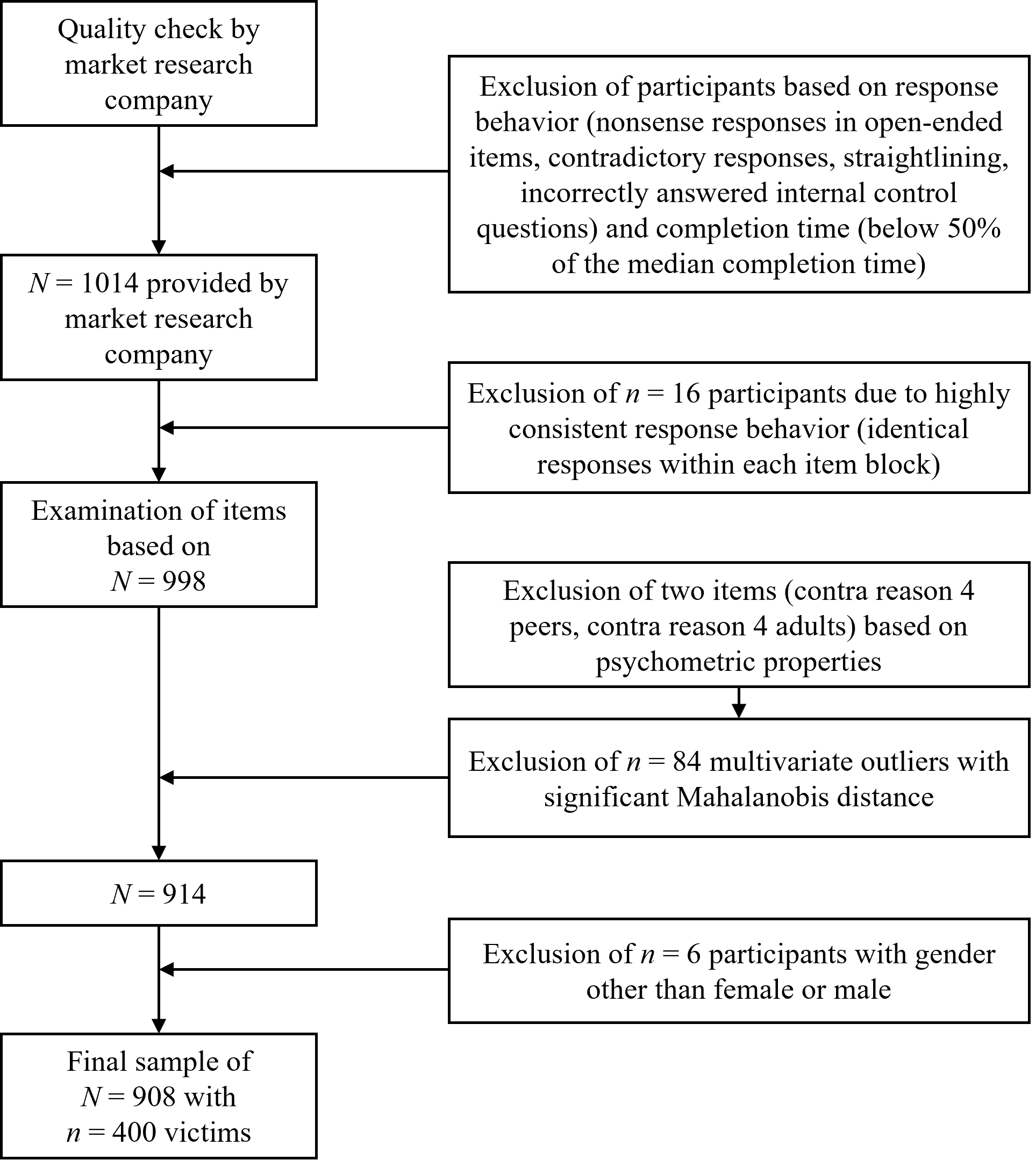


**SM Figure 2**


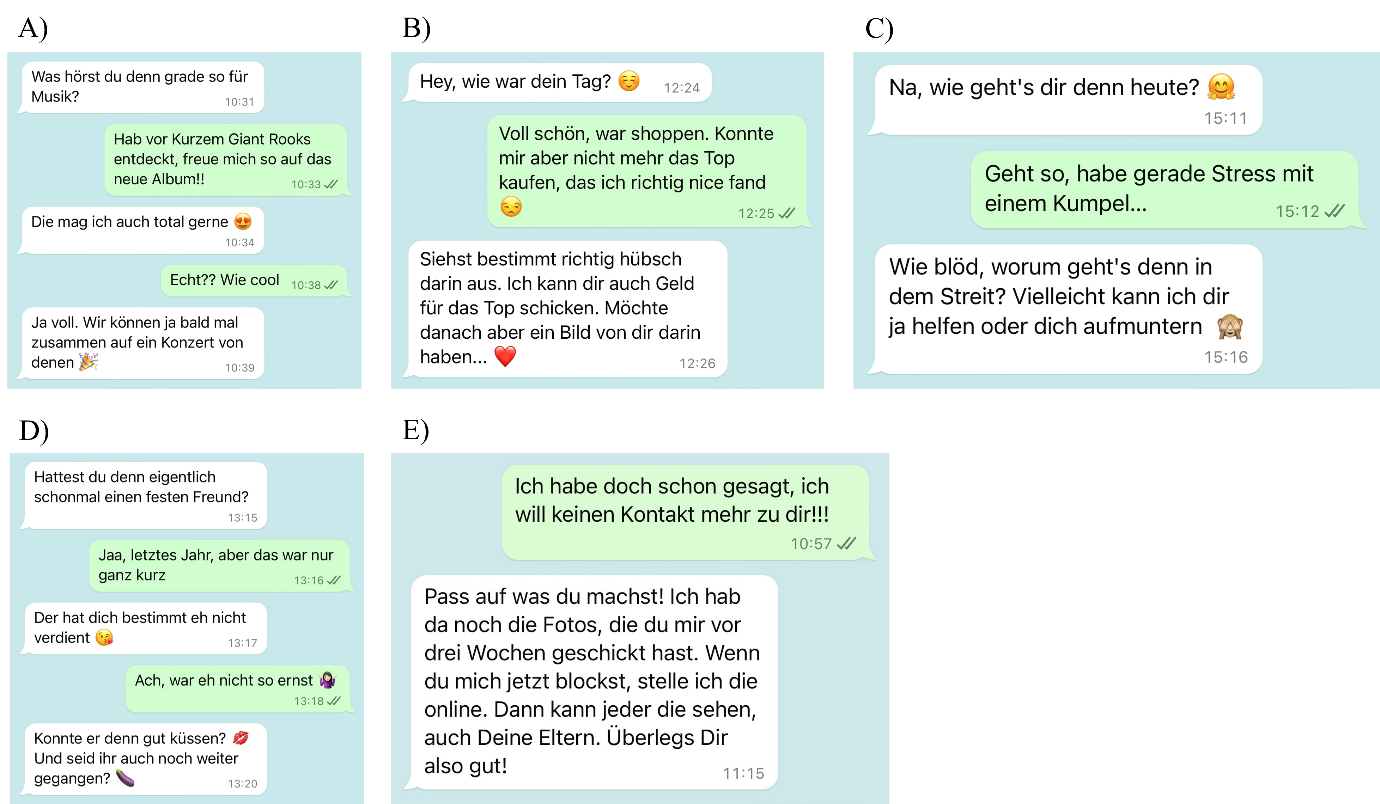
*Exemplary Chat Sequences Presented to Participants*

*Note*. A) Deception, B) Gift giving, C) Interest in victim environment, D) Sexualization, E) Aggression. Chat sequences were presented in an animated GIF-format.

**SM Figure 3**

*Measurement Models of Cybergrooming Victimization and Reasons For and Against Disclosure*


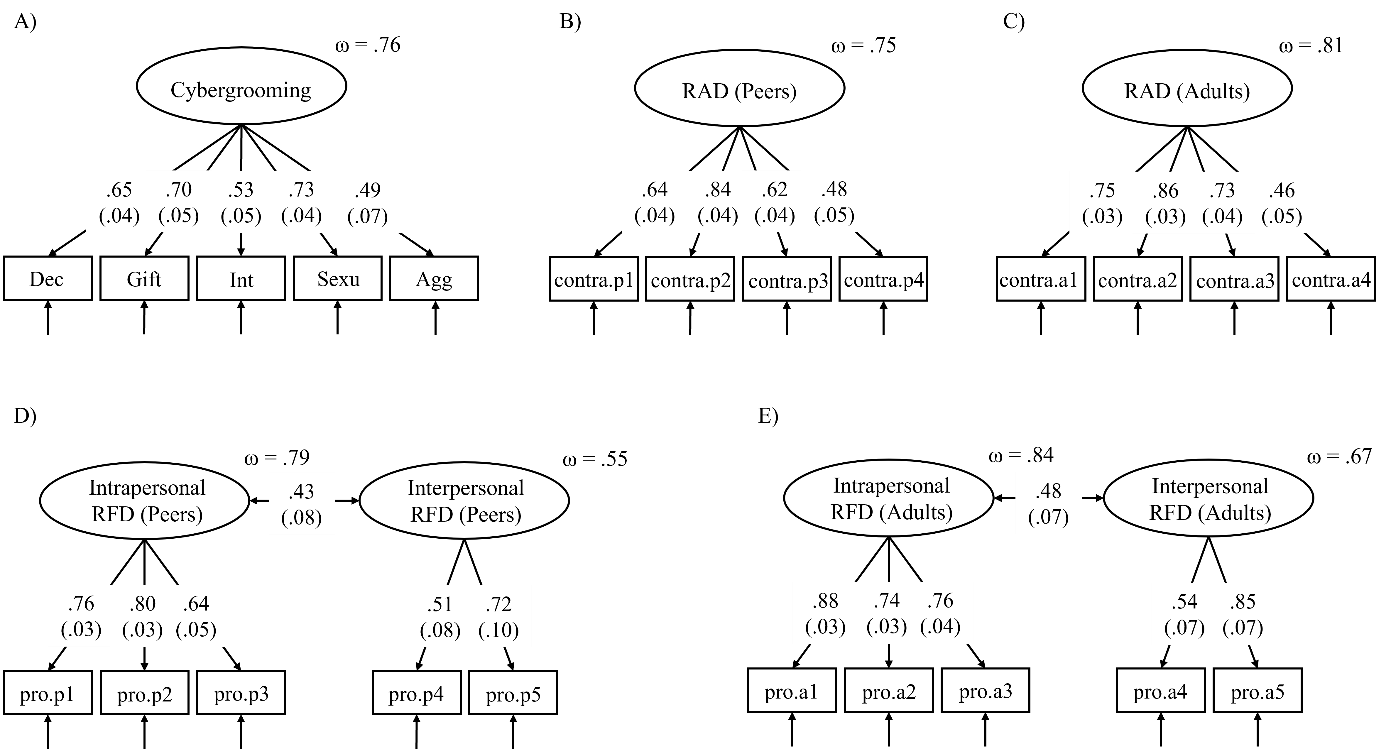


*Note*. A) Measurement model for cybergrooming victimization, B) Model for reasons against disclosure to peers, C) Model for reasons against disclosure to adults, D) Model for reasons for disclosure to peers, E) Model for reasons for disclosure to adults. Dec = Deception, Gift = Gift giving, Int = Interest in victim environment, Sexu = Sexualization, Agg = Aggression, RAD = Reasons against disclosure, RFD = Reasons for disclosure. All parameters are standardized. Figures in parentheses indicate standard errors.

**SM Table 1**

*Descriptive Statistics of All Items Based on n = 400 Victims*

| **Item** | **Description** | ***M* (*SD*) /**  **Yes/No (%)** | **Skew** | **Kurtosis** |
| --- | --- | --- | --- | --- |
| Such an adult person deceived me into believing that we have things in common or like the same things. | CG Deception | 0.96 (0.76) | 0.61 | 0.25 |
| Such an adult person offered me money or other things in exchange for photos or videos of myself. | CG Gift giving | 0.59 (0.77) | 1.31 | 1.40 |
| Such an adult person showed interest in how I was doing, if I was happy. | CG Interest | 1.21 (0.76) | 0.58 | 0.25 |
| Such an adult person asked me about my sexual experiences. | CG Sexualization | 0.66 (0.77) | 1.05 | 0.70 |
| Such an adult person sent me threatening or insulting messages. | CG Aggression | 0.53 (0.74) | 1.37 | 1.43 |
| How often have you had any contact with such an adult person? | CG Global | 1.29 (0.58) | 1.86 | 2.35 |
| Peers (e.g., friends or siblings) | Disclosure P | Yes: 72.8,  No: 27.2 | - | - |
| Adults (e.g., parents or teachers) | Disclosure A | Yes: 55,  No: 45 | - | - |
| So that the person stops contacting me. | Pro reasons P1 | 3.58 (1.21) | -0.53 | -0.61 |
| So that the person is punished. | Pro reasons P2 | 3.27 (1.29) | -0.18 | -1.06 |
| So that I feel better. | Pro reasons P3 | 4.24 (0.90) | -1.22 | 1.44 |
| Because I was asked about it. | Pro reasons P4 | 2.94 (1.16) | -0.13 | -0.83 |
| Because someone else has reported similar experiences. | Pro reasons P5 | 3.60 (1.14) | -0.62 | -0.35 |
| I was embarrassed. | Contra reasons P1 | 3.33 (1.29) | -0.43 | -0.89 |
| I was scared I wouldn't be believed. | Contra reasons P2 | 2.83 (1.37) | 0.04 | -1.27 |
| I was scared of being punished (e.g., Internet ban). | Contra reasons P3 | 2.50 (1.35) | 0.43 | -1.07 |
| I didn’t want the contact to end. | Contra reasons P4* | 1.80 (1.06) | 1.21 | 0.59 |
| The person blackmailed me, threatened me or told me not to tell anyone. | Contra reasons P5 | 2.14 (1.28) | 0.79 | -0.64 |
| So that the person stops contacting me. | Pro reasons A1 | 3.94 (1.21) | -0.94 | -0.14 |
| So that the person is punished. | Pro reasons A2 | 3.53 (1.31) | -0.46 | -0.96 |
| So that I feel better. | Pro reasons A3 | 4.11 (1.06) | -1.26 | 1.12 |
| Because I was asked about it. | Pro reasons A4 | 2.97 (1.26) | -0.11 | -1.00 |
| Because someone else has reported similar experiences. | Pro reasons A5 | 3.17 (1.35) | -0.23 | -1.09 |
| I was embarrassed. | Contra reasons A1 | 3.25 (1.42) | -0.39 | -1.18 |
| I was scared I wouldn't be believed. | Contra reasons A2 | 2.65 (1.37) | 0.26 | -1.18 |
| I was scared of being punished (e.g., Internet ban). | Contra reasons A3 | 2.67 (1.46) | 0.27 | -1.34 |
| I didn’t want the contact to end. | Contra reasons A4* | 1.83 (1.12) | 1.07 | -0.08 |
| The person blackmailed me, threatened me or told me not to tell anyone. | Contra reasons A5 | 2.14 (1.25) | 0.76 | -0.58 |

*Note*. CG = cybergrooming, P = peers, A = adults. *Item was excluded from analyses. “Such an adult person” (cybergrooming) and “the person” (reasons) were clarified in the instructions.
